# HSV-2 genome recognition by nuclear cGAS instigates IFN-β production and influences inflammasome activation during *de novo* infection in HFF cells

**DOI:** 10.3389/fimmu.2026.1717787

**Published:** 2026-04-13

**Authors:** Aditya Goel, Vatsala Kaul, Mir Faizan Ul Haq, Mohammad Akram, Nikita Chaudhary, David N. Everly, Mairaj Ahmed Ansari

**Affiliations:** 1 Department of Biotechnology, School of Chemical and Life Sciences, Jamia Hamdard, New Delhi, India; 2 Centre for Virology, School of Interdisciplinary Sciences and Technology, Jamia Hamdard, New Delhi, India; 3 Foundational Sciences and Humanities, Microbiology and Immunology, Chicago Medical School, School of Graduate and Postdoctoral Studies, Rosalind Franklin University of Medicine, Chicago, IL, United States

**Keywords:** cGAS and autophagy, nuclear cGAS recognizes herpesvirus genome, cGAS and DNA-damage-response, HSV-2, cGAS and IFI16 coordination, IFN-β, inflammasome, nuclear innate functions of cGAS

## Abstract

**Introduction:**

Type I interferon response, specifically, the cGAS-cGAMP-STING axis that results in IFN-β response, is well known for its complex roles early during viral infection. Previous reports suggest that HSV-1 DNA in Thp-1 cells and HIV-2 dsDNA in DCs and macrophages could be sensed by cGAS. The nuclear DNA sensor IFI16’s viral DNA sensing leads to its acetylation, cytoplasmic translocation and STING activation and inflammasome activation. Although cGAS is known to be associated with IFI16 in the nucleus, however, during HSV-2 infection, the role of nuclear cGAS in viral DNA sensing, inflammasome formation and type I IFN response remains unknown.

**Methods:**

In the current study, extensive investigation of the complex IFN-β responses elicited early during *de novo* HSV-2 infections in HFF cells is undertaken. The SiIFI16 and SicGAS treated HFF cells infected with HSV-2 demonstrate that cGAS senses nuclear herpes-viral DNA in an IFI16 dependent manner leading to nuclear cGAMP production.

**Results:**

These results unravel a novel nuclear cooperative role of cGAS and IFI16 and extend the cGAS DNA sensing and its enzymatic activity in the nucleus. IFI16 acetylation required for inflammasome complex formation is cGAS independent. The cGAS-pro-Caspase1 and cGAS-ASC interaction suggests plausible role of cGAS in inflammasome complex for Caspase-1 activation. The activated Caspase-1 interaction with cGAS was also observed. Further, the autophagy and DNA damage responses elicited during *de novo* HSV-2 infection are suggested.

**Discussion:**

The crosstalk of the type I interferon pathway with the inflammasome, autophagy and DNA damage response pathways suggests an intricate mechanism of inter-regulation at different stages and time points during infection, that might orchestrate a balanced and efficient immune response or facilitate viral immune evasion. Unique and dynamic post translational modifications of cGAS, namely acetylation and K-63 poly-ubiquitination, are observed, and are plausibly involved in cGAS regulation during HSV-2 infection.

## Introduction

The innate immune response is the first line of defense against invading pathogens and sterile insults in eukaryotes. The pattern recognition receptors (PRRs) are crucial components of this system, distributed at all possible sub-cellular locations, which recognize Pathogen Associated Molecular Patterns (PAMPs) and Damage Associated Molecular Patterns (DAMPs) and elicit appropriate immune responses. DNA sensors are a type of PRRs that recognize and respond to pathogen origin DNA in the nucleus and the cytoplasm as well as self-DNA in the cytoplasm. Earlier, it had been reported that among these DNA sensors, the cGAS (1), DDX41 (2), DAI (3), DHX9 and DHX36 (4) sensor molecules in the cytoplasm detect dsDNA and activate the STING-TBK1-IRF3 axis of type I IFN (IFN-β) response. IFI16 had been shown to sense transfected and fragmented HSV-1 DNA in the cytoplasm of macrophages (5). Recognition of cytoplasmic DNA by AIM2 had been shown to result in the activation of AIM2-ASC-pro-Caspase-1 inflammasomes resulting in activation of Caspase-1 and formation of proinflammatory interleukin-1β (IL-1β) and IL-18 cytokines.

During a viral infection, PRR stimulation leads to a rapid production of type I IFNs, a group of cytokines which are one of the first to be produced during innate antiviral response (1). Initial wave of type I IFN is dependent upon IRF3 phosphorylation and NFκβ activation (6–8), subsequent IRF7 phosphorylation increases type I IFN release. For instance, HSV-2 infection in a mouse model was reported to induce an initial wave of IFN-β production at 12 h post-infection, followed by both IFN-β and IFN-α production at 48 h post-infection (9, 10). Importantly, the early type I IFN production is crucial for induction of both an antiviral response within infected and target cells, as well as activation of innate immune cells. which ultimately control virus replication and activate the adaptive immune response, in order to both clear the infection and generate memory, so as to create a rapid response against future infections (11). Type I IFN upon binding to its receptor in target cells further induces expression of genes involved in preventing virus replication in these cells. From around 300 interferon stimulated genes (ISGs) which are upregulated, 51 were found to contribute to host defense, while others contributed to inflammation, signaling, transcription, and immunomodulation, among other activities (12, 13). Literature suggests that acute type I IFN production promotes antiviral responses beneficial for the host, while chronic type I IFN production can have a suppressive and deleterious effect on the immune response (14). Also, the timing and magnitude of type I IFNs produced can result in differing responses. Further, it has been hypothesized that along with promoting antiviral functions of innate immune cells, and later limiting these same functions, type I IFN limit damaging immune responses that can lead to tissue pathology and collateral damage (15). Therefore, we wanted to investigate the molecular mechanism and regulation of interferon-β production early during *de novo* herpesvirus infection in HFF cells.

Previously, we had demonstrated that IFI16, a multifunctional nuclear resident protein, is a sensor of herpes simplex virus type 1 (HSV-1), Kaposi’s sarcoma associated herpesvirus (KSHV), and Epstein-Barr virus (EBV) genomic dsDNA in the nucleus of B cells, human fibroblast (HFF) and endothelial (HMVEC-d) cells, and activates the inflammasome and IFN-β innate responses (16–19). IFI16, believed to interact with dsDNA in a sequence independent manner (20), modulates viral and cellular transcription by unknown mechanisms (21, 22). IFI16 also acts as a restriction factor for the lytic replication of human cytomegalovirus (HCMV), HSV-1 and KSHV (22–24). Recently, it was indicated that IFI16 binds to the most accessible regions of HSV-1 genome, and simultaneously globally reduces viral-DNA accessibility and expression of all viral proteins in each temporal gene class (25). We had shown that herpes viral genome recognition by IFI16 induces BRCA-1-dependent acetylation of IFI16 which is responsible for its association with ASC in the nucleus, nuclear to cytoplasmic translocation of the IFI16-ASC inflammasome complex, as well as IFI16’s association with STING in the cytoplasm resulting in the TBK1-IRF3-IFN-β response (26, 27).

Cyclic GMP-AMP synthase (cGAS), is optimally activated by a minimum 36 bp dsDNA, and can recognize various DNA viruses, certain retroviruses, and self-DNA in the cytoplasm (28, 29). Detection of DNA leads to a conformational change in cGAS which allows the entry of ATP and GTP into the catalytic pocket leading to cGAMP production which activates the STING-TBK1-IRF3-IFN-β response (1, 30). Although the detection of transfected or fragmented HSV-1 DNA by cGAS in the cytoplasm has been shown (5), direct association of cGAS with genomic herpesvirus dsDNA had not been established.

Although initially, cGAS was thought to be present in the cytoplasm, it was later reported to be present in the nucleus as well. Till now, there have only been two reports of nuclear cGAS being able to act as a sensor for viral DNA. In one of these, this sensing and activation of nuclear cGAS was shown against nuclear replicating DNA virus HSV-1, although only in THP-1 cells (31). The other report showed that upon infection with retro-virus HIV-2 in DCs and macrophages, the viral capsid in the nucleus is sensed by the NONO protein, which facilitates viral dsDNA sensing by nuclear cGAS, leading to its activation (32). Nevertheless, no studies till date have shown the direct interaction of cGAS with viral genome in the nucleus.

Orzalli et al. (33) reported that nuclear cGAS promotes IFI16 stability, hence, indirectly activates IFI16-mediated innate signaling in HSV-1 infected HFF cells. However, whether the cGAS interacts with viral DNA or not was not clear. Also, it was demonstrated that IFI16 could induce IRF3 activation for IFN-β production by activating STING via an unknown mechanism. Studies suggest that the pyrin domain of IFI16 mediates its interaction with cGAS, and only cGAS, but not IFI16, activates STING-TBK1-IRF-3 in HFF cells infected with 10 MOI of HSV-1 for 6h (34). This is not surprising as studies by us and others have shown that after 8h of infection, IFI16 is not detected in the HFF cells as it is targeted and degraded by HSV-1 immediate early protein ICP0 (18). In addition, a later study demonstrated that IFI16 is essential for DNA-activated cGAS-cGAMP-STING-TBK1 pathway for IFN-β in human macrophages (35). However, the nature and mechanism of IFI16 cooperation with cGAMP remained unclear. Nevertheless, this study supports our findings where we have shown that nuclear genome recognition induced acetylation of IFI16 leads to its cytoplasmic translocation and association with STING and induction of IFN-β response (26, 27, 36). Another study proposed that cGAS cooperates with IFI16 to activate the IFN-β response in human keratinocytes (34).

Whether cGAS also senses the herpes viral DNA in the nucleus and if so, whether it is dependent or independent of IFI16, and whether cGAS participates in nuclear inflammasome assembly, as well as the consequences of these events, are not known. Further, whether cGAS is involved in crosstalk with other innate pathways such as autophagy and DDR during HSV-2 infection in HFF cells is also not known.

Previous studies have also implicated the involvement of NLRP3 inflammasome during HSV-1 infection in different cell lines and murine models and most recently, during HSV-2 infection only in human macrophages. Upon activation, NLRP3 recruits ASC and pro-Caspase1, forming the inflammasome complex, leading to proteolytic cleavage of pro-Caspase-1 into its active form, which subsequently activates proinflammatory IL-1β and IL-18, which are then secreted out of the cell, thus amplifying inflammatory responses (37, 38). Active Caspase-1 also cleaves Gasdermin-D, producing active N-terminal fragments, which form pores in the membrane, further amplifying the inflammatory response, culminating into pyroptosis, a highly inflammatory form of programmed cell death (39, 40).

Further, previous reports on cGAS-STING axis-mediated NLRP3 inflammasome induction (41), and active Caspase-1-mediated cGAS suppression, during HSV-1 infection in different cells (42), prompted us to explore similar inter-regulation of the type I interferon and inflammasome pathways during HSV-2 infection in HFF cells.

Autophagy, a highly conserved catabolic process, functions during viral infections both as an antiviral defense mechanism and as a viral survival strategy (43). Consistently, studies on HSV-2 infection have implicated crucial role of modulation of autophagy by the virus for its survival (44, 45). During initiation of autophagy, the ULK-1 complex targets membrane structures at the phagophore formation site and triggers phagophore nucleation by recruiting the class III phosphatidylinositol 3-kinase complex I (PI3KC3-C1), which is comprised of a lipid kinase VPS34, a scaffold and putative protein kinase VPS15, a regulatory subunit BECN1, a pre-autophagosome assembly site (PAS) directing protein ATG14L (46–48) and an activator NRBF2. ULK-1 phosphorylation of PI3KC3-C1 components activates the complex, which produces phosphatidylinositol 3-phosphate (PI3P), increasing its local concentration for the recruitment of PIP3-binding autophagy proteins to the phagophore membrane. Nucleation is aided by trafficking of the ATG9-containing vesicles (48). Thereafter, during elongation and maturation, ATG12 conjugates with ATG5, followed by the recruitment of ATG16L1 to form a functional complex. Further, the microtubule-associated protein 1 light chain 3 (LC3) is cleaved by ATG4 at the C terminal, forming LC3-I. While ATG7 and ATG3 function as E1- and E2-like enzymes, the ATG12-5-16L1 complex functions as an E3-like enzyme, facilitating transfer of LC3-I to a phosphatidylethanolamine (PE) of the autophagosomal membrane (49). The PE-conjugated LC3B-I is known as LC3B-II. While LC3-II marks the formation of autophagosomal membrane, the efficiency of autophagic degradation is indicated by p62. Subsequently, the outer autophagosomal membrane fuses with a lysosome, forming an autophago-lysosome (50, 51).

Further, during HSV-2 infection in macrophages, type I IFN activated JAK-STAT pathway was reported to lead to autophagy induction, aiding in viral survival (45). However, it was not explored whether the two pathways are involved in crosstalk at the early stages of infection, in terms of viral DNA sensing and signaling activation.

Furthermore, during HSV-1 infection in different cell lines, cGAS was reported to interact with Beclin-1, leading to suppression of type I interferon pathway, while inducing autophagy, resulting in elimination of cytosolic pathogen DNA (52). However, whether there is role of nuclear cGAS in such interaction was not reported. STING has also been implicated to induce autophagy during HSV-1 infection (53). However, whether such crosstalk operates during HSV-2 infection in primary cell line HFFs has not been elucidated, which we explored here.

Herpesvirus infection and lytic replication trigger the host DNA damage response (DDR). The Homologous Recombination (HR) pathway, a type of DDR, is mediated by ataxia telangiectasia mutated protein (ATM), a PI3 kinase-like kinase, which is activated by phosphorylation upon sensing of a double-strand break by the MRN complex (Meiotic recombination protein 11 (MRE11), Radiation sensitive protein 50 (RAD50) and Nijmegen breakage syndrome protein 1 (NBS1)). pATM further acts on various substrates, including the histone variant H2AX, which upon phosphorylation is called γH2AX (54). Further, the γH2AX signal can spread as far as one to two megabases from the initial site of damage in an ATM- and Mediator of DNA Damage Checkpoint 1 (MDC1) -dependent manner (55). Subsequently, additional downstream effectors for repair are sequentially recruited to damage foci following ubiquitination of H2A-type histones by Ring finger protein 8 (RNF8) and RNF168 (56, 57). The herpesvirus lytic replication program selectively incorporates features of the DNA damage pathway, as indicated by studies on HCMV, EBV, HSV-1 and KSHV (58–62), which are required for efficient viral replication. Particular, early events such as ATM activation and H2AX phosphorylation are detected in cells. However, most studies have focused on DDR response during HSV-1 infection and then inferred for HSV-2 infection, therefore, we explored the DDR pathway during HSV-2 infection in HFF cells.

Further, a previous report of the interaction of cGAS and γH2AX due to DDR induced by etoposide, a genotoxic agent (63), prompted us to explore whether a similar crosstalk between type I IFN pathway and DDR occurs during HSV-2 infection.

The dynamic post translational modifications (PTMs) of cGAS regulates its functionality, and the same PTM at different residues can have different effects. Acetylation of cGAS has been reported to reduce upon HSV-1 and HCMV infection. Also, while various ubiquitination patterns of cGAS have been reported to have diverse effects on its functionality, its K-63 linked polyubiquitination has not been studied in case of HSV-2 infection.

While deciphering the innate responses elicited during *de novo* HSV-2 infection, we observed a very interesting biphasic IFN-β response. Further exploration revealed that cGAS is associated with IFI16 in the nucleus and the initial IFN-β response is dependent on IFI16 whereas the late response is an IFI16-independent but cGAS-dependent response. IFI16 is essential for cGAS’s ability to sense the nuclear input herpes viral DNA. Our studies uncover a complex cooperative role between IFI16 and cGAS during *de novo* HSV-2/1 infection and demonstrate that both IFI16 and cGAS are important for the elicitation of biphasic IFN-β response. We also found that cGAMP is produced in the nucleus early during infection to elicit a rapid early response, thus extending the innate function of cGAS and its enzymatic activity to the nucleus. The IFI16 acetylation, earlier reported to be indispensable for inflammasome complex formation was found to be cGAS independent. Moreover, the reduction of cleaved-Caspase-1 in cGAS knocked down cells suggests its plausible role in Caspase-1 activation in IFI16 and NLRP3 inflammasomes complex, early during HSV-2 infection. Further, we also observed the possible STING-NLRP3 axis of inflammasome activation. Furthermore, the dynamic cGAS-Beclin-1 and STING-LC3 interactions during infection suggest the crosstalk of type I IFN and autophagy pathways. Interestingly, we also observed the interaction of cGAS with γH2AX in the nucleus later during infection. The observed protein-protein interactions suggest an intricate crosstalk and inter regulation of the interferon pathway with other innate pathways during *de novo* HSV-2 infection, which could fine tune the innate immune response. We also report the dynamic acetylation and K-63 linked polyubiquitination of cGAS, representing another layer of regulation. Importantly, HSV-2 ICP27 was found to bind STING early during infection, highlighting the viral mechanisms employed for immune evasion.

## Materials and methods

### Cells

Vero (CRL-1586, ATCC, USA) and HFF-1 cells (SCRC-1041, ATCC, USA) were grown as described before (16–18). DMEM, penicillin/streptomycin and L-glutamine were from HiMedia Laboratories Pvt. Ltd., India. FBS was from Gibco (Thermo Fischer Scientific). Cells were routinely tested for mycoplasma and only mycoplasma free cells were used for experiments.

### Reagents

Protein A-Sepharose - 4B Fast Flow beads were from GE Healthcare Bio-Sciences Corp., Piscataway, NJ. Image-iT FX Signal Enhancer and Prolong Gold Antifade reagent with DAPI was from Life Technologies (Thermo Fischer Scientific).

### Antibodies

Mouse monoclonal anti-IFI16, mouse monoclonal anti-cGAS, mouse monoclonal anti-γ H2AX, mouse monoclonal anti-HSV-2 ICP27, mouse monoclonal anti-ASC, mouse monoclonal anti-IL-1β, mouse monoclonal anti-IL-18 antibodies were from Santa Cruz Biotechnology Inc., Santa Cruz, CA. Rabbit anti-BrdU antibody was from Rockland Inc., Gilbertsville, PA. Rabbit monoclonal anti-β-actin, rabbit anti-human polyclonal MAP1LC3A/B (N-Terminal) and rabbit anti-human polyclonal anti-Beclin-1 (N-Terminal) antibodies were from Bio-Rad. Rabbit anti-human TATA binding protein (TBP), rabbit antihuman Tubulin, rabbit anti-IRF-3, rabbit anti-pIRF-3, rabbit anti-STING, rabbit anti-p STING, rabbit anti-IFI16, rabbit anti-cGAS, rabbit anti-ASC, rabbit anti-K63 polyubiquitin antibodies were from CST, Danvers, MA. Rabbit anti-ATM, anti-pATM, rabbit polyclonal anti-Caspase-1 antibody and anti-cleaved-Caspase-1 antibody were from Affinity Biosciences, Cincinnati, Ohio. Rabbit polyclonal anti-Lamin-B1 antibody was Real-Gene Labs, Lake Forest, CA. Anti-rabbit and anti-mouse antibodies linked to horseradish peroxidase, Alexa Fluor-488, and -594 were from KPL Inc., Gaithersburg, MD, or Molecular Probes, Eugene, OR.

### HSV-2 and HSV-1

The HSV-2 (333 strain) and HSV-1 (KOS strain) were procured from ATCC. The virus was produced and titer determined by plaque assay on Vero cells as described previously (18).

### EdU and BrdU genome labelled HSV-2 and HSV-1

To generate 5-ethynyl-2'deoxyuridine (EdU) and 5-bromo-2-deoxyuridine (BrdU)-labelled infectious HSV-2 and EdU-labelled infectious HSV-1, we added EdU or BrdU labelling reagent (Life Technologies) to the culture medium at 8h, 24h and 48h post infection in a 1:100 (v:v) ratio (from the supplied stock). Flasks with media containing BrdU were kept in darkness during incubation to avoid photolysis of BrdU residues. Collection and purification of labelled virus were carried out as described before (27).

### HSV-2 and HSV-1 infection

HFF cells were starved for 2h washed and infected with HSV-2 or HSV-1 at a multiplicity of infection (MOI) of 1 PFU/cell (~25 genome copies/cell) in serum-free DMEM for different times, washed with PBS, and incubated in DMEM supplemented with 2% FBS for different time points.

### Si-RNA transfection

All Si-RNA oligonucleotides for IFI16 and cGAS were from Santa Cruz Biotechnology, Inc. Primary HFF cells were transfected with Si-RNA using a Neon transfection system (Invitrogen) according to the manufacturer’s instructions. Briefly, sub-confluent cells detached from culture flasks were washed once with PBS and resuspended in buffer R (Invitrogen) at a density of 1X10^7^ cells/ml. 10 μl of the cell suspension was gently mixed with control Si-RNA or 100 pmol of target specific Si-RNA and then microporated at room temperature using a single pulse of 1,350 V for 30 ms. Thereafter, cells were distributed into pre-warmed complete medium and placed at 37 °C in a humidified 5% CO2 atmosphere. At 48h post-transfection, cells were infected with HSV-2 as described earlier and incubated for 2, 4, 8 or 24 hours, whole cell lysates using RIPA or cytoplasmic/nuclear extract buffers were isolated, knockdown efficiency evaluated by western blotting, and then subjected to co-IP and western blotting (26).

### IFN-β ELISA

HFF cells were starved and not infected or infected with HSV-2 for 4, 8 or 24 hours. Culture supernatants were centrifuged and subjected to ELISA for detection of IFN-β, performed as per manufacturer’s instruction. Briefly, culture supernatants and standards were incubated in the precoated wells for 1h, washed with the washing buffer provided in the kit and probed with IFN-β antibody for 1h. These wells were washed, incubated with HRP tagged antibodies for 1h, washed, incubated with substrate (TMB) for 15 min and the reaction was terminated with a stop solution. After 5 min, readings were taken and calculations done using a standard curve (26). The IFN-β levels in pg/ml were statistically analyzed using GraphPad Prism. Error bars represent S.D.

### Immunofluorescence microscopy assay

HFF cells grown on 8 well chamber glass slides for 48h were serum-starved in the presence or absence of inhibitors for 2h, washed and then either left uninfected or infected with HSV-2 (1 PFU/cell) for 2h. Cells were washed with PBS, incubated in complete medium for various time points, washed, fixed in 4% paraformaldehyde for 10 min and permeabilized with 0.2% Triton X-100 for 5 min. Cells were washed and blocked with Image-iT FX signal enhancer (Invitrogen) for 20 min at RT, and incubated with specific antibodies diluted in 2% BSA for 2h at 37 °C. After washing, cells were incubated with the appropriate Alexa-Fluor conjugated appropriate secondary antibodies for 1h at 37 °C, washed, mounted in DAPI, imaged with Thermo EVOS M7000 (26) and analyzed with CellProfiler cell image analysis software (64). The Pearson’s correlation coefficient among the red and green channels was calculated through the software over the entire image as a measure of the co-localization of two proteins. The DAPI channel was used to identify the nuclei. The cells were identified by employing appropriate methods to expand the region through the red and/or green channels wherever applicable. The cytoplasmic region was identified as the difference between the cellular and nuclear boundaries. The Pearson’s correlation was measured in the nuclear and/or cytoplasmic regions as applicable. Where required, the total count of foci in the red or green channel representing protein localization in the nuclear region was calculated through the software over the entire image. Pre-designed pipelines for each application in the software were optimized as per recommendations (64). The Pearson’s correlation coefficients or total foci counts were statistically analyzed using GraphPad Prism.

### *In situ* proximity ligation assay microscopy

A DuoLink PLA kit from Sigma-Aldrich was used to detect protein–protein interactions as per manufacturer’s protocol. Cells were infected with HSV-2 (1 PFU/cell; ~25 genome copies/cell), fixed and permeabilized as described in the IFA section and blocked with DuoLink blocking buffer for 30 min at 37°C in a humid chamber. These cells were incubated with target specific primary antibodies diluted in DuoLink dilution buffer. After washing, the cells were incubated for another 1h at 37°C with species specific PLA probes (PLUS and MINUS) under hybridization conditions and in the presence of 2 additional oligonucleotides to facilitate hybridization of PLA probes if they were in close proximity (<40 nm). A ligation mixture and ligase were then added to join the two hybridized oligonucleotides to form a closed circle. Amplification solution was added to generate a concatemeric product extending from the oligonucleotide arm of the PLA probe. Finally, a detection solution consisting of fluorescently labelled oligonucleotides was added, and the labelled oligonucleotides were hybridized to the concatemeric products. The signal was detected as a distinct fluorescent dot in the Texas red or GFP green channel and analyzed by fluorescence microscopy (26). Cells were selected at random. In a particular region, two cells were analyzed and three such regions were analyzed. The number of PLA dots per cell were counted manually and statistically analyzed using GraphPad Prism. Error bars represent S.D.

### Co-immunoprecipitation

HSV-2 infection induced protein-protein interactions were evaluated by co-IP experiments using equal amounts (20 µg) of WCL as well as cytoplasmic and nuclear lysates. The lysates were first incubated for 2h with 15 μl of Protein A-Sepharose - 4B Fast Flow beads and then the pre-cleared lysates were incubated for 2h with immunoprecipitating antibody at 4°C. The immune complexes were captured using 15 μl of beads, washed 4 times with lysis buffer, 3 times in PBS, boiled with SDS-PAGE sample buffer, resolved by 10% SDS-PAGE, and then subjected to western blotting (26).

### Western blot analysis

The whole cell protein lysates (WCL) from uninfected and HSV-2 infected cells were prepared using RIPA buffer supplemented with protease inhibitor cocktail and PMSF (Sigma). Cells were incubated on a rocker at 4°C for 15 min and sonicated at 60 amplitude three times, with pulses of 30 seconds on and 30 seconds off. Lysates were clarified by centrifugation for 15 min at 4 °C at 15000 rcf. The nuclear and cytoplasmic extracts were prepared following the protocol standardized in our lab. Equal amounts of samples (20 µg) were resolved by 10-20% SDS-PAGE, subjected to western blot, immunoreactive bands developed using SuperKine West Pico Plus or Femto Maximum Sensitivity Chemiluminescence Kit (Abbkine), and the bands scanned and densitometric quantification was performed using a ChemiDoc and ImageLab software (Bio-Rad) (26). Normalization and fold change calculations were performed wherever indicated.

### Genome pull down assay

The EdU-labelled viral genome (chromatin) pull-down assay was performed as described earlier (27). Briefly, HFF cells untreated or treated with SiIFI16 or SicGAS for 48h, either left uninfected or infected with unlabeled or EdU labelled HSV-2 (10 PFU/cell) or HSV-1 (10 PFU/cell) for 2h were cross-linked using 1% formaldehyde at 4 °C for 10 min in the dark. The unreacted formaldehyde was quenched by adding 0.125 M glycine for 10 min at 4 °C, cells were harvested, permeabilized with 0.1% Triton X-100 for 10 min and washed with PBS. A Click reaction was performed by sequential addition of 10 mM (+)-sodium-L-ascorbate, biotinTEZ azide (0.1 mM) and copper (II) sulphate (2 mM) for 30 min in the dark followed by adding 1% BSA and 0.5% Tween 20 for 10 min. Biotin was linked to EdU labelled genome by Click reaction. The soluble proteins were isolated in 500 μl CL lysis buffer (50 mM HEPES, pH 7.8, 0.25% Triton X-100, 0.5% NP-40, 150 mM NaCl, 10% glycerol plus protease inhibitors) for 10 min at 4°C and centrifuged at 300xg. The pellet containing chromatin-protein complexes was washed with wash buffer (10 mM Tris-HCL, pH 8.0, 0.5 mM DTT, 200 mM NaCl) at 4°C for 10 min and then resuspended in 500 μl RIPA buffer (10 mM Tris-HCl, pH 8.0, 0.1% NaDeoxycholate, 0.1% SDS, 1% Triton X-100 and 140 mM NaCl plus protease inhibitor cocktail) and chromatin was sheared by sonication. The sonicated extract was clarified by centrifugation (15,000xg) for 10 min at 4 °C and 1 mg of the extract was used for pull down using 50μl of streptavidin magnetic beads. Beads with bound complexes were subjected to reverse protein-DNA cross-linking and proteins were eluted in 1X Laemmli sample buffer (95 °C for 10 min) for immunoblotting. To purify DNA, the complexes were eluted from beads in elution buffer (0.1 M NaHCO3 and 1% SDS) and cross-linking was reversed by treating with 0.1 mg/ml RNase A and 0.3M NaCl for 30 minutes at 37 °C and then incubated at 65 °C for 2h with 0.1 mg/ml Proteinase K. Eventually, DNA was column purified using a Qiagen DNA extraction kit as per manufacturer’s instructions (27).

### cGAMP ELISA

The cytoplasmic and nuclear fractions of HFF cells uninfected or infected with HSV-2 for 4 or 24 hours were subjected to ELISA for detection of 2’3’ cGAMP present according to the manufacturer’s instructions (Cayman 2’3’ cGAMP ELISA Kit). Briefly, fractions were diluted using Immunoassay buffer C. Controls, standards and samples were added in the precoated wells, followed by the addition of the 2’3’ cGAMP-HRP Tracer to the indicated wells and probing with the 2’3’ cGAMP ELISA polyclonal anti-serum and then incubated overnight. Thereafter, wells were washed with the washing buffer provided in the kit and incubated with substrate (TMB) for 30 min and the reaction was terminated with a stop solution. After 5 min, readings were taken and calculations done using a standard curve. The cGAMP levels in pg/ml were statistically analyzed using GraphPad Prism. Error bars represent S.D.

### Statistical analysis

Results are expressed as means ± SD of at least three independent experiments (n≥3). The p value was calculated using an Ordinary One-way ANOVA or Two-way ANOVA as applicable. In all tests, p<0.05 was considered statistically significant.

## Results

### The biphasic IFN-β production was observed during *de novo* HSV-2 infection in HFF cells

**Figure 1:**
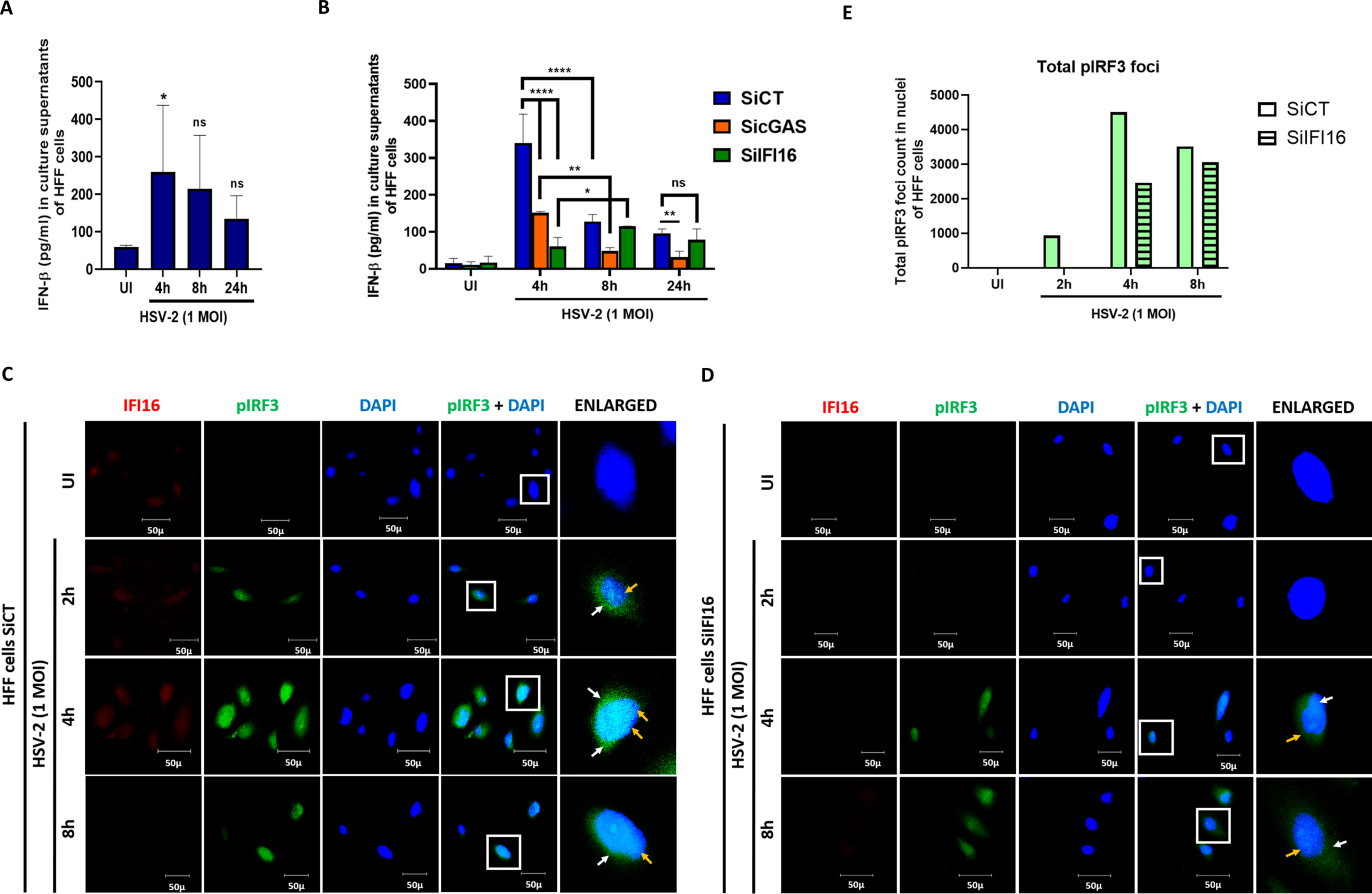
*De novo* HSV-2 infection in HFF cells induces the biphasic production of IFN-β. **(A)** Interferon-β levels in culture supernatants (CS) of HFF cells uninfected or HSV-2 (1 MOI) infected at 4, 8 and 24 h.p.i. was quantitated by ELISA. **(B)** Interferon-β levels in CS of HFF cells electroporated with SiCT, SicGAS or SiIFI16 and left uninfected or infected with HSV-2 (1 MOI) for 4, 8 and 24h was quantitated by ELISA. The data represent the mean of three determinants ± S.D. and are representative of three independent experiments with similar observation. *p<0.05, **p<0.01 and ****p<0.0001. NS, Non-significant. Control **(C)** or IFI16 knockdown **(D)** HFF cells were infected for 2, 4 or 8h with HSV-2 (1 MOI), processed for IFA, reacted with anti-IFI16 and anti-pIRF3 antibodies followed by Alexa Fluor-594 and Alexa Fluor-488 secondary antibodies and DAPI (blue) (**Table 1**). The boxed areas are enlarged. Translocation of pIRF3 from cytoplasm to nucleus is suggested. 40X magnification. **(E)** Total pIRF3 foci in nuclei of the cells counted over the entire image as a measurement of amount of pIRF3 localized in the nuclei.

Recently, IFI16 and cGAS have been shown to coordinate to induce innate response during HSV-1 infection in macrophages (33). Here we are trying to decipher the level of coordination between IFI16 and cGAS early during HSV-2 *de-novo* infection. *De novo* HSV-2 infection of 1 MOI in HFF cells leads to the production and secretion of IFN-β in HFF cells, with highest levels at 4 h.p.i., declining at 8 h.p.i. and the further reducing at 24 h.p.i. (**Figure 1A**). In order to assess the specific time-dependent biphasic production of IFN-β, we tried to decipher the plausible roles of IFI16 and cGAS. The cGAS or IFI16 in HFF cells were knocked down through SiRNA transfection (**Figure 1B**). The IFI16 knockdown reduces approximately 7 fold IFN-β production in comparison to SiCT (scrambled SiRNA) at 4 h.p.i. On the other hand, at 4 h.p.i. cGAS knockdown slightly hampered IFN-β levels compared to SiCT (approximately 2.2 fold). This indicates that IFI16 alone, contributes to the major fraction of IFN-β levels, and cGAS alone contributes only to a small fraction of IFN-β production at 4 h.p.i. Further, reduced IFN-β levels at 4 h.p.i. in cGAS knockdown cells compared to control indicate towards the importance of cGAS in stabilizing IFI16 for optimal production of IFN-β. Previous studies have established that at later time points of infection, viral E3 ubiquitin ligase ICP0 degrades IFI16 (65). Consistently, at 8h post HSV-2 infection, levels of IFN-β were lower by approximately 2.77 fold in SiCT HFF cells than in SiCT cells at 4 h.p.i. Further, in cGAS knockdown cells, IFN-β levels at 8 h.p.i. were significantly reduced than SiCT cells (approximately 2.68 fold). And furthermore, IFN-β production in cGAS knockdown cells was significantly lower (approximately 3.25 fold) at 8 h.p.i. than 4 h.p.i., seemingly owing to IFI16 being degraded. Interestingly, however, the IFN-β levels in IFI16 knockdown cells at 8h were significantly higher (approximately 2.2 fold) than that at 4h and almost comparable to SiCT cells at 8 h.p.i. (approximately 1.1 fold change only), indicating that absence of IFI16 does not have much effect on IFN-β production at 8 h.p.i. Importantly, IFN-β levels in IFI16 knockdown were approximately 2.46 fold higher than in cGAS knockdown cells at 8 h.p.i. Taken together, the reason for this observation is clarified, indicating that at 8h post HSV-2 infection in HFF cells, overall cGAS innate function is reduced, however, at this time point only cGAS but not IFI16 is contributing in IFN-β production. Similar pattern of IFN-β production were observed at 24 h.p.i., with the levels in all three cases being slightly lower than that at 8 h.p.i. In conclusion, IFI16 induces IFN-β within 4 h.p.i. and cGAS is responsible for later time point, however, overall type I IFN reduces at 8 h.p.i and 24 h.p.i. time point.

**Table 1:** List of antibodies used in the study.

Antibody	Species	Source	Catalogue no.
Acetylated lysine	Rabbit polyclonal	CST, Danvers, MA	9441
AIM2 (D5X7K)	Rabbit monoclonal	CST, Danvers, MA	12948
ASC(B-3)	Mouse monoclonal	Santa Cruz Biotechnology Inc., Santa Cruz, CA	Sc-514414
ASC/TMS1(E1E3I0)	Rabbit monoclonal	CST, Danvers, MA	13833
ATG5	Rabbit polyclonal	Affinity Biosciences, Cincinnati, Ohio	DF6010
ATM	Rabbit polyclonal	Affinity Biosciences, Cincinnati, Ohio	AF4119
B-actin	Mouse monoclonal	Invitrogen, Carlsbad, CA	MA5-15739
Beclin-1 (N-terminal)	Rabbit polyclonal	Bio-Rad Laboratories, Inc., Hercules, CA	AHP1009
BrdU (BU-1)	Mouse monoclonal	Invitrogen, Carlsbad, CA	MA3-071
B-tubulin (9F3)	Rabbit monoclonal	CST, Danvers, MA	2128
Caspase-1	Rabbit polyclonal	BioVision, Milpitas, CA	3019-100
Caspase 1 Ab	Rabbit polyclonal	Affinity Biosciences, Cincinnati, Ohio	AF5418
Cleaved caspase-1(Asp 296), p20Ab	Rabbit polyclonal	Affinity Biosciences, Cincinnati, Ohio	AF4005
cGAS (c-1)	Mouse monoclonal	Santa Cruz Biotechnology Inc., Santa Cruz, CA	Sc-515802
cGAS (DID3G)	Rabbit monoclonal	CST, Danvers, MA	15102
GASDERMIN D (E504N)	Rabbit monoclonal	CST, Danvers, MA	69469
HSV-1/2 Gb (10B7)	Mouse monoclonal	Santa Cruz Biotechnology Inc., Santa Cruz, CA	Sc-56987
HSV-1/2 ICP27(H1142)	Mouse monoclonal	Santa Cruz Biotechnology Inc., Santa Cruz, CA	Sc-69806
IL-1B(11E5)	Mouse monoclonal	Santa Cruz Biotechnology Inc., Santa Cruz, CA	Sc-52012
IFI16(D8B5T)	Rabbit	CST, Danvers, MA	14970
IFI16(IG7)	Mouse monoclonal	Santa Cruz Biotechnology Inc., Santa Cruz, CA	Sc-8023
IRF3(D83B9)	Rabbit monoclonal	CST, Danvers, MA	4302
K63 polyubiquitin (P37)	Rabbit polyclonal	CST, Danvers, MA	58395
Lamin-B1	Rabbit monoclonal	Real-Gene Labs, Lake Forest, CA	4220032
MAP1LC3A/B (N-terminal)	Rabbit polyclonal	Bio-Rad Laboratories, Inc., Hercules, CA	AHP2167
NLRP3 (D4D8T)	Rabbit monoclonal	CST, Danvers, MA	15101
Phospho-ATM (Ser 1893)	Rabbit Polyclonal	Affinity Biosciences, Cincinnati, Ohio	AF8100
p-HistoneH2AX (Ser 139)	Mouse monoclonal	Santa Cruz Biotechnology Inc., Santa Cruz, CA	Sc-517348
Phospho-IRF3 (Ser396) (D601M)	Rabbit monoclonal	CST, Danvers, MA	29047
Phospho-STING (Ser366) (D7C3S)	Rabbit monoclonal	CST, Danvers, MA	19781
STING (D2P2F)	Rabbit monoclonal	CST, Danvers, MA	13647
TBP (D59CH) XP	Rabbit monoclonal	CST, Danvers, MA	44059
ANTI-MOUSE IgG(H+L) F(ab’)2 Fragment ALEXA FLUOR 488 Conjugate secondary	Goat	MA Molecular Probes, Invitrogen, Carlsbad, CA	4408S
ANTI-RABBIT IgG(H+L) F(ab’)2 Fragment ALEXA FLUOR 594 Conjugate secondary	Goat	Molecular Probes, Invitrogen, Carlsbad, CA	8889S
Anti-mouse IgG, HRP Linked secondary	Goat	Genei Laboratories, Bangalore, Karnataka	114068001A
Anti-rabbit IgG, HRP Linked secondary	Goat	Genei Laboratories, Bangalore, Karnataka	114038001A

In mock transfected and uninfected HFF cells, pIRF3 is not observed, while at 2 h.p.i., it is seen both in the cytoplasm as well as in the nucleus, representing its cytoplasm to nuclear translocation (**Figure 1C**). Further, its amount increases to maximum at 4h, present in both the cytoplasm (yellow arrow) and the nucleus (white arrow). At 8h, pIRF3 is seen to remain only in the nucleus, and its amount is reduced. These observations are in line with the secreted IFN-β detected through ELISA (**Figure 1B**).

In IFI16 knocked down and uninfected HFF cells, pIRF3 is not observed, as expected (**Figure 1D**). However, at 2 h.p.i., it is still not observed, indicating the crucial role of IFI16 in phosphorylation of IRF3, and thus the IFN-β production. At 4 h.p.i., however, it is observed in both the cytoplasm and the nucleus, indicating the activity of a pathway other than the IFI16-STING pathway leading to IFN-β production. At 8 h.p.i., pIRF3 is still observed, although lower in amount, but present in the cytoplasm as well as the nucleus. **Figure 1E** is a graphical representation of the total count of pIRF3 in HFF cells under conditions stated in **Figure 1C** and **Figure 1D**. These observations indicate that in the absence of IFI16, IRF3 is still phosphorylated through another pathway which operates at 4 hours and 8 hours post infection, with highest pIRF3 being present at 4h, thereafter slightly decreasing at 8h (**Figures 1C–E**). These observations support the pattern of pIRF3 expression observed through western blot as discussed in the following result, in line with the secreted IFN-β detected through ELISA.

### HSV-2 infection induces IFI16-cGAS and IFI16-STING-dependent IRF3 activation as well as modulation of cGAS post translational modifications

**Figure 2:**
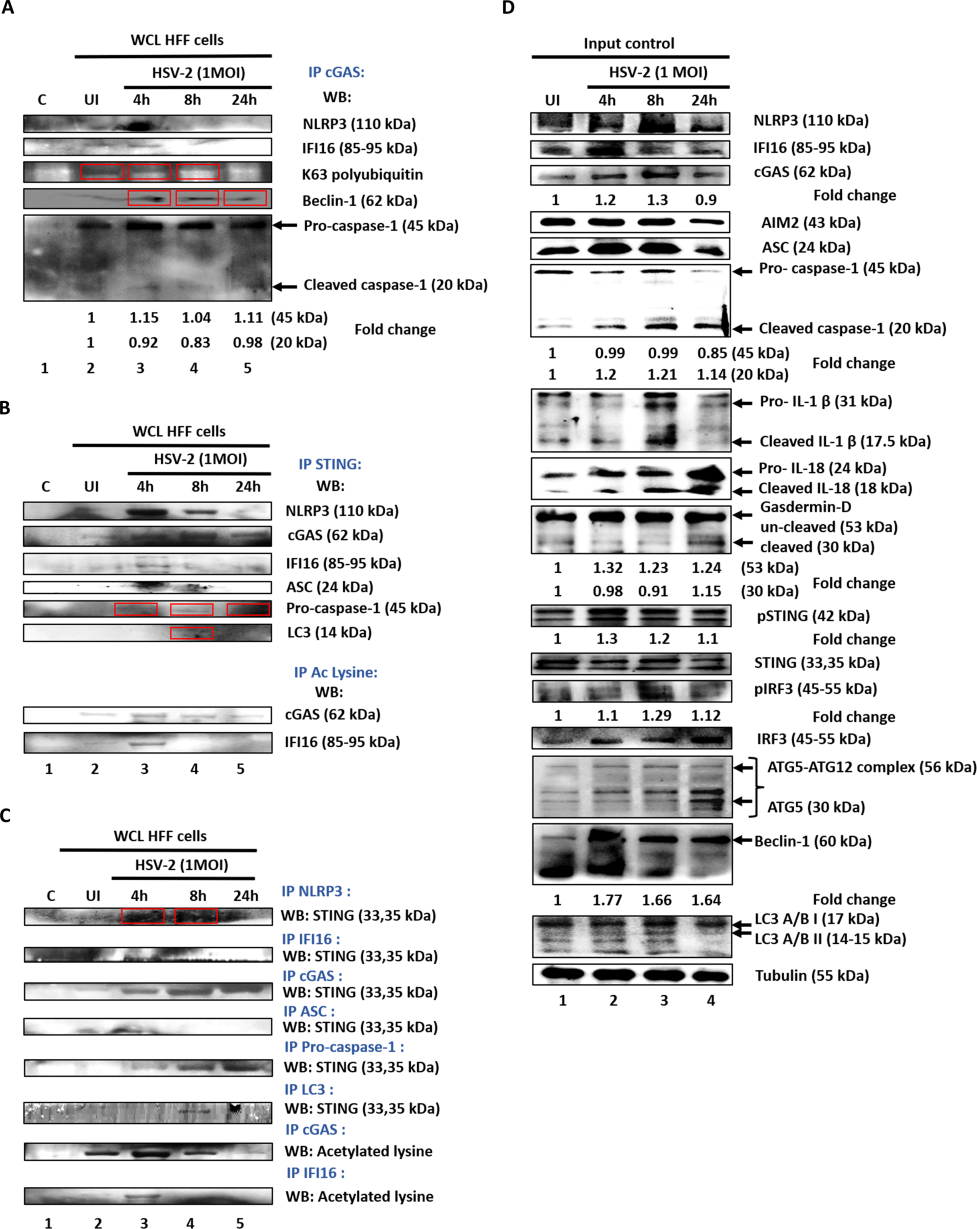
*De novo* HSV-2 infection induces IFI16-cGAS and IFI16-STING-dependent IRF3 activation, and the crosstalk of type I interferon pathway with inflammasome and autophagy pathways, as well as dynamic ubiquitination and acetylation of cGAS. HFF cells were left uninfected or infected with HSV-2 at 1 MOI for 4, 8 or 24h. **(A)** Equal amounts of whole cell lysate proteins were IPed with anti-cGAS antibody and western blotted for NLRP3, IFI16, K-63 linkage-specific polyubiquitin, Beclin-1 and Caspase-1 (pro and cleaved) (**Table 1**). Fold changes are included at the bottom of the respective blots. **(B)** Equal amounts of whole cell lysate proteins were IPed with anti-STING antibody and western blotted for NLRP3, IFI16, cGAS, ASC, pro-Caspase-1 and LC3 IIA/B. Equal amounts of whole cell lysate proteins were IPed with anti-Acetylated Lysine antibody and western blotted for IFI16 and cGAS. Red boxes are used to identify bands. **(C)** Equal amounts of whole cell lysate proteins were IPed with antibodies against the proteins found to interact with STING and western blotted for STING or IPed with antibodies against cGAS or IFI16 and western blotted for Acetylated Lysine. **(D)** Levels of total NLRP3, IFI16, cGAS, AIM2, ASC, Caspase-1 (pro and cleaved), IL-1β (pro and cleaved), IL-18 (pro- and cleaved), Gasdermin-D (pro and cleaved), STING, pSTING, IRF3, pIRF3, ATG5, Beclin-1, LC3 IIA/B and Tubulin (as loading control) were detected with their respective antibodies. Fold changes are included at the bottom of the respective blots.

In order to understand the regulation of cGAS for biphasic IFN-β production, we tried to understand it’s interactions with other innate molecules. cGAS interacts with IFI16 at 4 h.p.i. (**Figure 2A**), in line with the established role of cGAS in stabilizing IFI16 for efficient viral dsDNA binding and eliciting antiviral responses (33). Interestingly, we observed that cGAS also undergoes K-63-linked polyubiquitination in uninfected condition and at 4 h.p.i., which thereafter significantly reduces at 8 h.p.i., and is absent at 24 h.p.i. (**Figure 2A**). Recently, this PTM mediated by MARCH8 enzyme was shown to inhibit the DNA binding ability and enzymatic activity of cGAS, suppressing innate immunity against HSV-1 infection (65). However, ours is the first report of this particular PTM of cGAS during HSV-2 infection in HFF cells, and its exact role and mechanism will be elucidated in further studies. We also found that cGAS interacts with Beclin-1 at 4h and 8h post HSV-2 infection (**Figure 2A**), similar to an earlier report of cGAS-Beclin-1 interaction during HSV-1 infection, which was shown to induce autophagy and simultaneously suppress cGAS activity (52). Interestingly, we found that cGAS also interacts with both pro-Caspase-1 as well as cleaved-Caspase-1 (**Figure 2A**). The interaction of cGAS with pro-Caspase-1 was observed in uninfected condition, and was increased to a maximum at 4h post HSV-2 infection (1.15 fold), slightly reduced at 8h (1.04 fold) and again increased at 24h (1.11 fold). Whereas, the interaction of cGAS with cleaved-Caspase-1 was observed in uninfected condition, slightly reduced at 4 h.p.i. (0.92 fold), further reduced at 8h (0.83 fold), and then increased at 24h (0.98 fold). Previously, during HSV-1 infection, it has been reported that activated Caspase-1 targets cGAS, resulting in the suppression of the cGAS-STING axis of type I IFN response (42). Thus, our observations warrant further investigation into the plausible negative regulation of cGAS activity by the activated inflammasome during HSV-2 infection.

However, we allowed our line of thought to extend in the reverse direction too, that is, whether cGAS could also regulate the inflammasome response. Since it is known that cGAS interacts with IFI16 at 4 h.p.i., we tried to find out whether it also interacts with NLRP3, the other inflammasome sensor known to operate during *de novo* HSV-1 infection in HFF cells (18), and during *de novo* HSV-2 infection in macrophages (67). Interestingly, we found that cGAS indeed interacts with NLRP3 at 4 h.p.i. (**Figure 2A**), an observation which has not been reported yet.

The interaction of IFI16 and STING was observed only at 4h post HSV-2 infection, indicating activation of the IFI16-STING pathway for IFN-β production (**Figure 2B**). Interestingly, cGAS was found to interact with STING. However, this interaction was observed even in uninfected condition, although very less, and was slightly increased at 4h post HSV-2 infection and substantially increased to a maximum at 8h and thereafter decreased at 24h. Interaction pattern suggests the role of IFI16-STING pathway only at 4h in IFN-β production, in line with the ELISA results (**Figure 1B**). Whereas, a distinct cGAS-STING signaling might be operational at 4, 8 and 24h but maximally at 8h, complementing the observations of ELISA (**Figure 1B**). Together, they suggest how the IFN-β production during early HSV-2 infection is orchestrated by IFI16-STING pathway, and later is taken over by the cGAS-STING pathway.

Interestingly, STING was also found to interact with NLRP3 at 4h post HSV-2 infection, and this interaction was lower at 8h and almost absent at 24h (**Figure 2B**). We also observed an interaction of STING with ASC at 4h post infection, which was then almost absent at 8h (**Figure 2B**). This pattern also corresponded to the STING-NLRP3 interaction pattern. Importantly, STING was seen to interact with pro-Caspase-1 only after HSV-2 infection, and this interaction was slight at 4 h.p.i., thereafter increasing at 8 and 24 h.p.i. (**Figure 2B**). Together, these interactions of STING with NLRP3 (sensor for inflammasome), ASC (adaptor) and pro-Caspase-1 (effector) indicate that it is in complex with the complete NLRP3 inflammasome, and corroborating with previously reported STING-mediated NLRP3 inflammasome activation during HSV-1 infection in other cell lines and in mice (41).

However, the STING-NLRP3 axis is observed maximally at 4h, reduced at 8h and diminished at 24h, suggesting NLRP3 inflammasome activation at these time points (**Figure 2B**). Interestingly, at 4h post infection, since STING has been found to interact with IFI16 as well as cGAS, in addition to NLRP3, it raises a question about the dynamic individual and relative contribution of the two DNA sensors towards the STING-NLRP3 axis of inflammasome activation. This would be clarified by further knockdown studies. While IFI16-STING interaction is seen only at 4h, cGAS-STING interaction is seen to increase from uninfected condition to 4h, where STING-NLRP3 interaction is observed. At 8h, although cGAS-STING interaction further increased, however, the STING-NLRP3 interaction decreases, suggesting that factors other than the cGAS-STING interaction might indeed be involved in deciding the STING-NLRP3 interaction. Similarly, at 24h, although a reduced cGAS-STING interaction still exists, STING-NLRP3 interaction is almost absent.

Importantly, STING was also seen to interact with LC3, only at 8h post infection (**Figure 2B**). Previously, during dsDNA stimulation and HSV-1 infection, STING has been implicated to induce autophagy by interacting with LC3 (53).

Acetylation of IFI16 was observed very minimally in uninfected condition, increasing to a maximum at 4h post infection, and then being almost absent at 8h and 24h (**Figure 2B**), and the IFI16 acetylation at 4 h.p.i. compliments the observed IFI16-STING interaction, in line with the previously established mechanism, wherein recognition of herpesvirus genome by IFI16 in the nucleus leads to IFI16 acetylation, which is crucial for IFI16 inflammasome assembly in the nucleus and its cytoplasmic translocation, as well as IFI16-mediated activation of STING in the cytoplasm (26). Reverse - immmunoprecipitation also showed similar interaction patterns as in **Figure 2B** (**Figure 2C**), further validating these observations.

### *De novo* HSV-2 infection induces inflammasome and autophagy responses

Uninfected or HSV-2 infected HFF whole cell lysates were western blotted for the inflammasome components (**Figure 2D** input control). The hallmark of inflammasome activation, cleaved-Caspase-1, is least in uninfected condition, increases at 4h post infection (1.2 fold) and is maximum at 8h (1.21 fold), thereafter only slightly decreasing at 24h (1.14 fold), even though the pro-Caspase-1 level at 24h is comparatively lower (0.85 fold). Gasdermin-D increases from uninfected condition up to 4h post infection, remaining stable at 8h, thereafter slightly decreasing at 24h. Cleaved-Gasdermin-D is maximum at 24h (1.15 fold). The levels of pro-IL-1β are almost similar in uninfected condition and 4h post infection, thereafter increasing to a maximum at 8h and then decreasing at 24h. Similar pattern is observed for cleaved-IL-1β as well as cleaved-IL-18 levels. Cleavage of pro-IL-1 β into its mature form, which is maximum at 8h, but decreases at 24h, could also be attributed to the already low level of pro-IL-1β at this time point.

Together, these observations indicate the activation of Caspase-1 and production of inflammatory cytokines IL-1β and IL-18 as well as Gasdermin-D cleavage, culminating in pyroptosis. Interestingly, availability of cleaved-Caspase-1 at 24h in absence of IFI16 indicates inflammasome activation by another sensor.

It has been reported that during *de novo* infection of HSV-1 in HFF cells, in the early stages (2h to 4h), the virus induced the activation of the IFI16 and NLRP3 inflammasomes and maturation of IL-1β. However, the immediate early protein ICP0 specifically targeted IFI16 for rapid proteasomal degradation at later times post-infection (18).

Beclin-1 levels are significantly enhanced at 4h post HSV-2 infection (1.77 fold), thereafter slightly reducing at 8h (1.66 fold) and 24h (1.64 fold). Further, ATG-5 levels increase up to 24h post infection, while the band indicating the ATG5-ATG12 complex also shows higher intensity, suggesting towards the elongation and maturation phase of the autophagy process. Furthermore, the levels of LC3-II increase to a maximum at 8h post infection, suggesting the formation of the autophagosomal membrane, while its levels thereafter drastically reduce at 24h.

### Dynamic K-63 polyubiquitination of cGAS in the nucleus and cytoplasm and interactions of cGAS with inflammasome, autophagy and DDR proteins upon *de novo* HSV-2 infection in HFF cells

**Figure 3:**
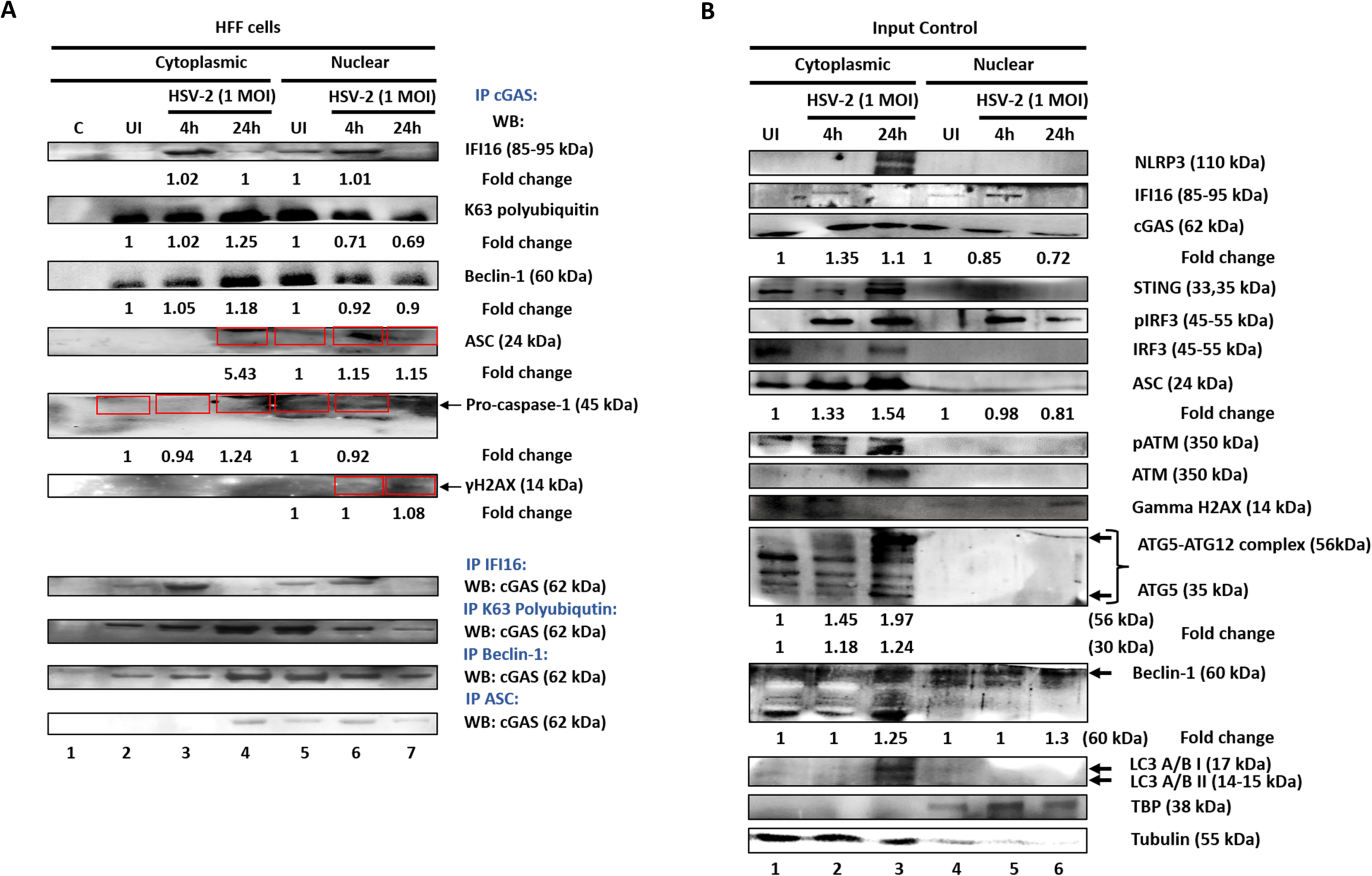
Cytoplasmic and nuclear distribution of cGAS and other innate molecules. **(A)** HFF cells were left uninfected or infected with HSV-2 at 1 MOI for 4 or 24h. Equal amounts of cytoplasmic and nuclear fractions were immuno-precipitated with anti-cGAS antibody and western blotted for IFI16, K-63 linked polyubiquitin, Beclin-1, ASC, Caspase-1 and γH2AX. Equal amounts of cytoplasmic and nuclear fractions were immuno-precipitated with antibodies against the proteins found to interact with cGAS and western blotted for cGAS. Red boxes are used to identify bands. Fold changes are included at the bottom of the respective blots. **(B)** Input controls are represented, with Tubulin and TBP as purity and loading controls. Fold changes are included at the bottom of the respective blots.

Interestingly, we observed K-63 polyubiquitination of cGAS, in both the nuclear and cytoplasmic fractions, in uninfected condition as well as early and late time points of HSV-2 infection (**Figure 3A**). In uninfected condition, K-63 polyubiquitination of nuclear cGAS is higher than that of cytoplasmic cGAS. At 4 hours post HSV-2 infection, K-63 polyubiquitination of nuclear cGAS decreases substantially (0.71 fold), while that of cytoplasmic cGAS increases slightly (1.02 fold). At 24 h.p.i., K-63 polyubiquitination of nuclear cGAS further decreases (0.69 fold), while that of cytoplasmic cGAS further increases (1.25 fold). Importantly, this is the first report of this PTM of cGAS in the nucleus and cytoplasm in HFF cells, which shows altered patterns during HSV-2 infection. Interestingly, together with the pattern of cGAS ubiquitination observed in the whole cell lysate (**Figure 2A**), these observations warrant further investigations to decipher the mechanism governing the dynamic pattern of the K-63 polyubiquitination of cGAS, and its distribution in different subcellular locations during HSV-2 infection in HFF cells.

We also observed that the interaction of Beclin-1 with cGAS occurred both, in the nucleus and in the cytoplasm (**Figure 3A**). Importantly, this interaction was seen in the uninfected condition, at low level in the cytoplasm, but at significantly high level in the nucleus. At 4 h.p.i., while this interaction in the cytoplasm increased (1.05 fold), contrarily, the interaction in the nucleus slightly decreased (0.92 fold). While at 24 h.p.i., the interaction further substantially increased in the cytoplasm (1.18 fold), it further decreased in the nucleus (0.9 fold). Since previously HSV-1 infection has been reported to lead to Beclin-1 interaction with cGAS (52), it is interesting to see their interaction increasing in the cytoplasm and decreasing in the nucleus during HSV-2 infection in HFF cells.

Interestingly, cGAS was found to interact with ASC in the nucleus, and this interaction was only slight in the uninfected condition, increased at 4 hours post HSV-2 infection (1.15 fold), and then remained consistent at 24 h.p.i., while in the cytoplasm their significant interaction was observed only at 24 hp.i. (5.43 fold) (**Figure 3A**).

We also observed that cGAS interacts with pro-Caspase-1 in both the nucleus as well as the cytoplasm, in the uninfected condition, which reduced at 4 hours post HSV-2 infection (0.92 fold in nucleus and 0.94 fold in cytoplasm). At 24 h.p.i., their interaction was not observed in the nucleus but was observed to increase in the cytoplasm (1.24 fold) (**Figure 3A**). Together, these observations (**Figures 2A**, **3A**) suggest that cGAS dynamically interacts with the inflammasome sensors (IFI16 and NLRP3), adaptor (ASC) and effector (pro-Caspase-1), suggesting that cGAS is in complex with the completely formed inflammasome. Importantly, since in the cytoplasm, interaction of cGAS with pro-Caspase-1 in the cytoplasm is seen in uninfected condition and 4 h.p.i. and 24 h.p.i. while that of cGAS and ASC in the cytoplasm is seen only at 24 h.p.i. (**Figure 3A**), it could also be speculated that ASC comes later at 24 h.p.i. for the formation of the complete inflammasome complex in the cytoplasm.

However, since the interaction of pro-Caspase-1 with cGAS stays till 8 and 24 h.p.i. as seen in the WCL (**Figure 2A**) and as seen in the cytoplasm at 24 h.p.i. (**Figure 3A**), while with IFI16 and NLRP3 is not observed at 8 and 24 h.p.i. (**Figures 2A**, **3A**), the exact kinetic mechanism of the formation and activity of these inflammasomes in complex with cGAS needs to be elucidated, which will be undertaken in or further studies.

Interestingly, we also found a very slight interaction of cGAS with the phosphorylated H2AX (γH2AX) protein in the nucleus at 8 hours post HSV-2 infection which increased at 24 h.p.i. (1.08 fold) (**Figure 3A**). This observation correlates to a previous study, wherein cGAS was found to interact with γH2AX, a known marker of double stranded breaks in DNA (63), and the interaction increased in response to DNA damage induced via genotoxic agents such as etoposide. The phosphorylation of H2AX S139 was required for the direct interaction of H2AX with cGAS, through cGAS C-terminal region (amino acids 213–522) (Kd = 176.8 nM), which facilitated the recruitment of cGAS to DNA-damage sites. Reverse - immunoprecipitation also showed similar interaction patterns as in **Figure 3A** upper panel (**Figure 3A** lower panel), further validating these observations.

### Cytoplasmic and nuclear distribution of molecules involved in type I IFN, inflammasome, autophagy and DDR pathways during *de novo* HSV-2 infection

Uninfected or HSV-2 infected HFF cytoplasmic and nuclear extracts were western blotted for the indicated antibodies against proteins involved in the type I interferon and inflammasome responses (**Figure 3B**).

In uninfected condition, IFI16 is observed in the nucleus (**Figure 3B**). At 4h post HSV-2 infection, it is reduced in the nucleus, while it is clearly increased in the cytoplasm, indicating its translocation from the nucleus to the cytoplasm. At 24 h.p.i., IFI16 is not seen in the nucleus as well as the cytoplasm, consistent with the fact that it is degraded by ICP0 in the nucleus (65), while in the cytoplasm it is seen in higher amount than at 4h. In uninfected condition, cGAS is present in both, the nucleus as well as cytoplasm. At 4 h.p.i., its levels reduce in the nucleus (0.85 fold) while increase in the cytoplasm (1.35 fold), thereafter, further reducing at 24 h.p.i. in the nucleus (0.72 fold) as well as in the cytoplasm (1.1 fold). STING is observed in the cytoplasm at all time points, highest at 24 h.p.i. It is also seen in nucleus in uninfected condition, consistent with reports of STING being present in nuclear membrane. IRF3 is seen only in cytoplasm. pIRF3 is seen in cytoplasm at 24h post infection (**Figure 3B**).

NLRP3 being a cytoplasmic sensor of inflammasome pathway, is seen in cytoplasm, at 24h post infection. The adaptor protein ASC is seen in cytoplasm, with levels increasing from uninfected to 4h.p.i. (1.33 fold) and 24h.p.i. (1.54 fold) (**Figure 3B**). This suggests that the IFI16 inflammasome operates at early time point and later NLRP3 inflammasome takes over.

Uninfected or HSV-2 infected HFF cytoplasmic and nuclear extracts were western blotted for the indicated antibodies against proteins involved in the autophagy pathway (**Figure 3B**). Beclin-1 levels were found to be increasing in the nucleus (1.3 fold) as well as in the cytoplasm (1.25 fold) at 24 h.p.i. infection (**Figure 3B**). Nuclear localization of beclin-1 was also reported in other studies (68). ATG5 as well as ATG5-ATG12 are observed only in cytoplasm. ATG-5 levels increase at 4 h.p.i. (1.18 fold) and 24 h.p.i. (1.24 fold). ATG5-ATG12 levels also follow similar pattern (1.45 fold at 4 h.p.i. and 1.97 fold at 24 h.p.i.). LC3-I is seen to increase from uninfected condition to 4h and 24h post infection in the cytoplasm, showing only slight conversion to LC3-II only at 24h. These observations indicate that the autophagy process culminates towards the late stage at 24h post HSV-2 infection in HFF cells.

Uninfected or HSV-2 infected HFF cytoplasmic and nuclear extracts were western blotted for the indicated antibodies against proteins involved in the DDR (**Figure 3B**). ATM is seen in cytoplasm, increasing from uninfected condition towards 4h post HSV-2 infection and is maximum at 24h in the cytoplasm. Phosphorylated ATM, though also observed in uninfected condition, was observed to be significantly increased at 4h and 24h post infection in the cytoplasm (**Figure 3B**). Phosphorylated H2AX was observed to be present in uninfected condition, distinctly increasing in the nucleus from uninfected condition towards 4h and 24h post infection, while it is also seen in the cytoplasm in uninfected condition and at 4h, thereafter decreasing at 24h. Our observations indicate that upon HSV-2 infection, the expression of ATM increases at 4h and 24h, which is then phosphorylated, further leading to phosphorylation of H2AX to γH2AX, which is present in the nucleus maximally at 24h.

### *De novo* HSV-2 infection in HFF cells induces IFI16-cGAS interaction at 4h in the cytoplasm

**Figure 4:**
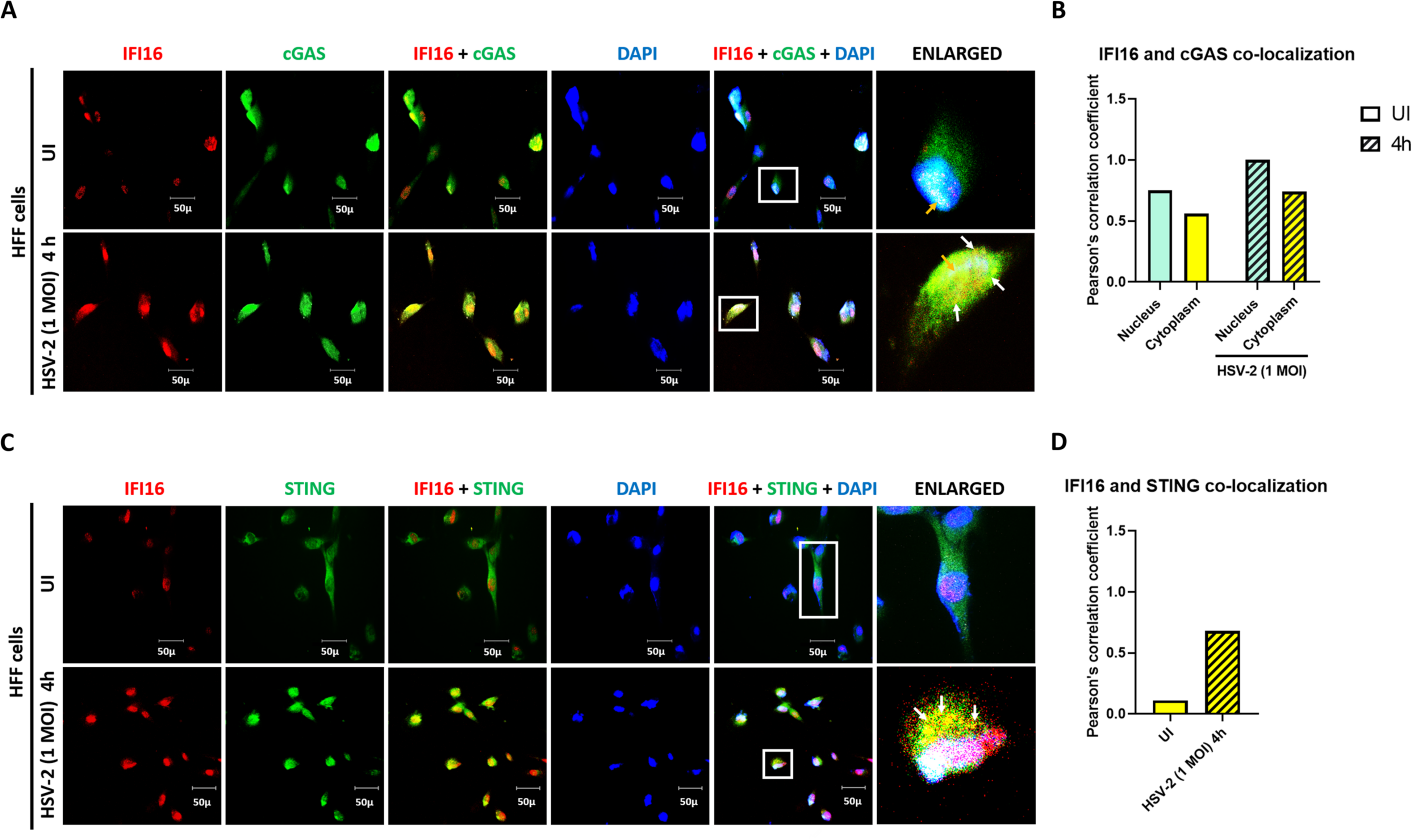
Cytoplasmic and nuclear distribution of cGAS-IFI16 and IFI16-STING complexes in HSV-2 infected HFF cells. **(A)** HFF cells were infected for 4h with HSV-2 (1 MOI), processed for IFA, reacted with anti-IFI16 and anti-cGAS antibodies followed by Alexa Fluor-594/488 secondary antibodies and DAPI (blue). The boxed areas are enlarged. Co-localization of IFI16 with cGAS in the cytoplasm (yellow spots, white arrows) and in the nucleus (white spots, yellow arrow). 40X magnification. **(B)** Pearson’s correlation coefficient among the red and green channels over the entire field as a measurement of co-localization of IFI16 and cGAS in the nucleus and the cytoplasm. **(C)** HFF cells were infected for 4h with HSV-2 (1 MOI), processed for IFA, reacted with anti-IFI16 and anti-STING antibodies followed by Alexa Fluor-594/488 secondary antibodies and DAPI (blue). The boxed areas are enlarged. Co-localization of IFI16 with STING in the cytoplasm (yellow spots, white arrows). 40X magnification. **(D)** Pearson’s correlation coefficient among the red and green channels over the entire field as a measurement of co-localization of IFI16 and STING in the cytoplasm.

In uninfected HFF cells, IFI16 (red) is present in the nucleus. cGAS (green) is present in the cytoplasm, and can also be seen in the nucleus (**Figures 4A**). At 4h post HSV-2 infection, the amount of IFI16 in the nucleus substantially increases, and it is also seen in the cytoplasm, indicating nuclear to cytoplasmic export. IFI16 and cGAS are observed to interact (yellow puncta) in uninfected condition in the nucleus (blue), as evident from the white color (yellow arrow). At 4 h.p.i., the IFI16-cGAS interaction (yellow puncta) increases in the nucleus (white color, yellow arrow) and their interaction is also visible in the cytoplasm (yellow puncta, white arrow) (**Figures 4A, B**). This supports the observation that cGAS interacts with IFI16 upon infection (**Figures 2A**, **3A**) and that this complex is also present in the cytoplasm.

### HSV-2 induces IFI16-STING interaction at 4h in the cytoplasm

In uninfected HFF cells, IFI16 (red) is present in the nucleus, while STING (green) is present in the cytoplasm (**Figures 4C, D**). At 4h post HSV-2 infection, the amount of IFI16 in the nucleus substantially increases and it is also seen in the cytoplasm, indicating nuclear to cytoplasmic translocation. The amount of STING in the cytoplasm also increases substantially. IFI16 and STING are seen to interact in the cytoplasm as evident from the yellow puncta, indicating the activation of the IFI16-STING pathway. The interaction observed here supports the interaction observed by co-immunoprecipitation using STING antibody (**Figures 2B, C**).

### K-63 polyubiquitination and acetylation of cGAS during *de novo* HSV-2 infection in HFF cells

**Figure 5:**
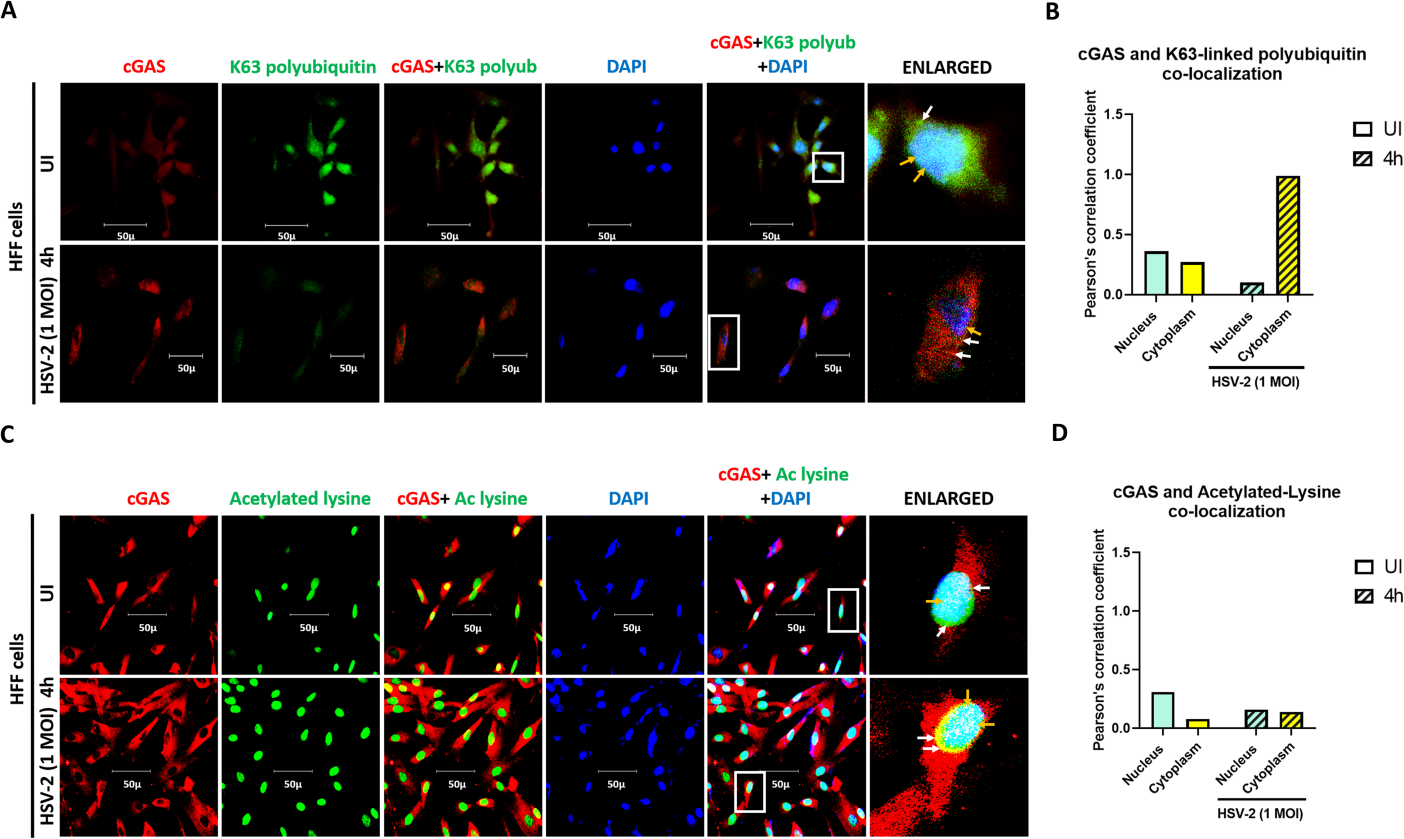
Cytoplasmic and nuclear distribution of cGAS having K-63 polyubiquitin and acetylation in HSV-2 infected HFF cells. **(A)** HFF cells were infected for 4h with HSV-2 (1 MOI), processed for IFA, reacted with anti-cGAS and anti-K-63 linkage-specific polyubiquitin antibodies followed by Alexa Fluor-594/488 secondary antibodies and DAPI (blue). The boxed areas are enlarged. Co-localization of cGAS with K-63 linkage-specific polyubiquitin in the cytoplasm (yellow spots, white arrows) and in the nucleus (white spots, yellow arrows). 40X magnification. **(B)** Pearson’s correlation coefficient among the red and green channels over the entire field as a measurement of co-localization of cGAS and K-63 linkage-specific polyubiquitin in the nucleus and the cytoplasm. **(C)** HFF cells were infected for 4h with HSV-2 (1 MOI), processed for IFA, reacted with anti-cGAS and anti-acetylated lysine antibodies followed by Alexa Fluor-594/488 secondary antibodies and DAPI (blue). The boxed areas are enlarged. Co-localization of cGAS with lysine acetylation in cytoplasm (yellow spots, white arrows) and in the nucleus (white spots, yellow arrows). 40X magnification. **(D)** Pearson’s correlation coefficient among the red and green channels over the entire field as a measurement of co-localization of cGAS and lysine acetylation in the nucleus and the cytoplasm.

In uninfected cells, cGAS (red) is present in the cytoplasm, and can also be seen in the nucleus, while K-63-linked polyubiquitin (green) is observed to be distributed in the cytoplasm as well as in the nucleus of the cell (**Figure 5A**). The interaction of cGAS and K-63-linked polyubiquitin is observed both in the cytoplasm (yellow spots, white arrows) as well as in the nucleus (white spots, yellow arrow) and further, the intensity of yellow spots is comparatively more in the nucleus than in the cytoplasm. At 4h post HSV-2 infection, more distinct yellow spots are observed in the cytoplasm (white arrow), whereas the intensity of white color in the nucleus (yellow arrow) is reduced compared to that in the uninfected condition (**Figures 5A, B**). Together with the immuno-precipitation results (**Figures 2A**, **3A**), these observations suggest that cGAS undergoes K-63-linked polyubiquitination, in the uninfected condition, at higher levels in the nucleus than in the in the cytoplasm, and upon HSV-2 infection, K-63-linked polyubiquitination of cGAS in the nucleus decreases, while it increases in the cytoplasm, with increasing time points post infection.

In uninfected cells, cGAS (red) is seen in the cytoplasm and can also be seen in the nucleus, while lysine acetylation (green) is seen only in the nucleus (**Figure 5C**). The lysine acetylation of cGAS is observed in the nucleus (white puncta) (**Figures 5C, D**). At 4h post HSV-2 infection, a slight increase in the intensity of white puncta is observed, indicating increase in acetylation of nuclear cGAS. Together with the immuno-precipitation result, these observations suggests that cGAS in the nucleus undergoes acetylation, in uninfected condition, which increases at 4h post HSV-2 infection (**Figures 2B, C**, **5C, D**). Thereafter, it reduces at later time points (**Figure 2B**). The exact role and mechanism of this PTM of cGAS will be elucidated in our further studies.

### cGAS interacts with autophagy protein Beclin-1 at early as well as later time points during *de novo* HSV-2 infection

**Figure 6:**
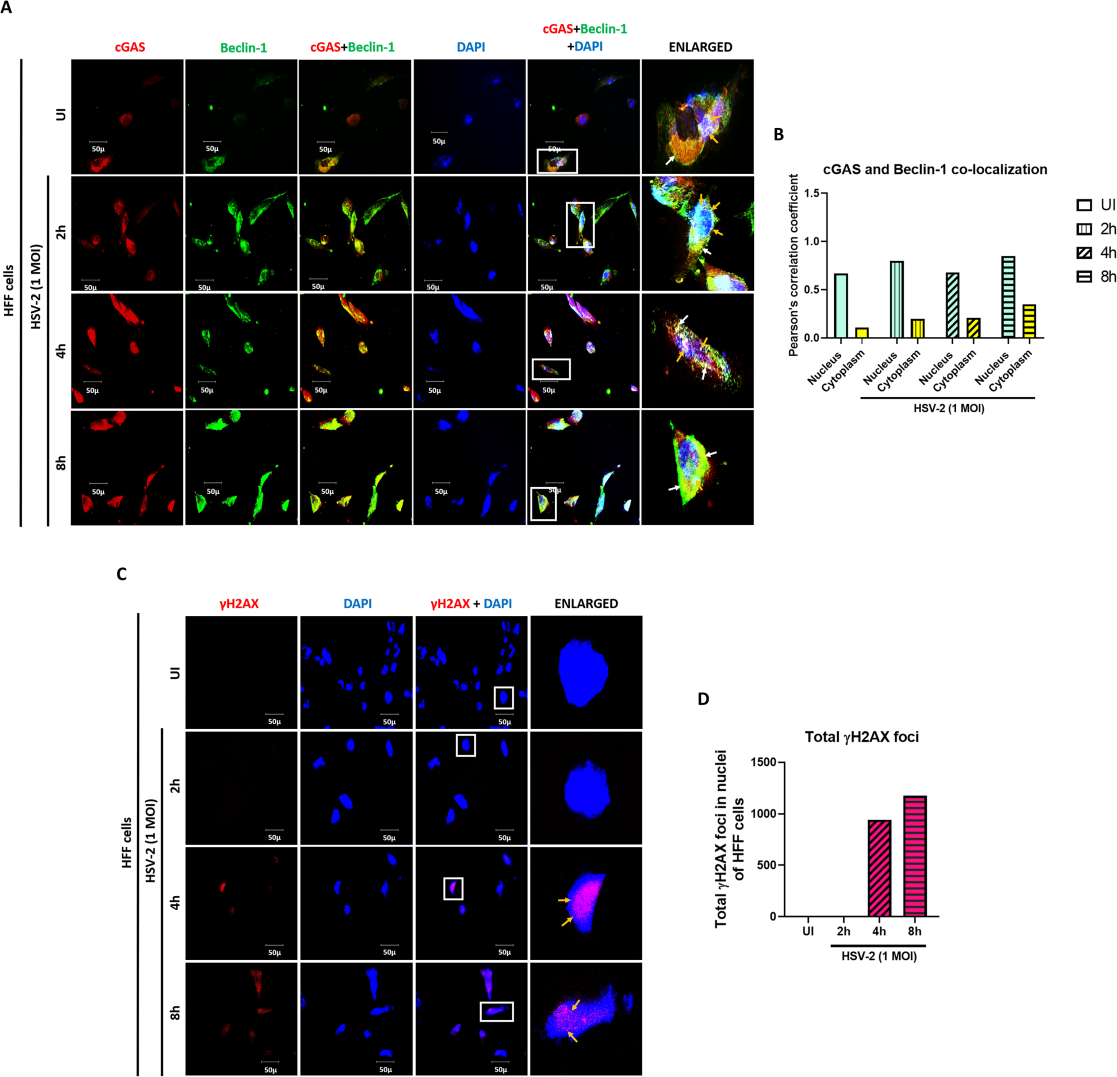
Cytoplasmic and nuclear distribution of cGAS in complex with Beclin-1 and amount of γH2AX in the nucleus post HSV-2 infection. **(A)** HFF cells were infected for 2, 4 or 8h with HSV-2 (1 MOI), processed for IFA, reacted with anti-cGAS and anti-Beclin-1 antibodies followed by Alexa Fluor-594/488 secondary antibodies and DAPI (blue). The boxed areas are enlarged. Co-localization of cGAS with Beclin-1 in the nucleus (white spots, yellow arrows), at the nuclear membrane, perinuclear space and in the cytoplasm (yellow spots, white arrows). 40X magnification. **(B)** Pearson’s correlation coefficient among the red and green channels over the entire field as a measurement of co-localization of cGAS and Beclin-1 in the nucleus and the cytoplasm. **(C)** HFF cells were infected for 2, 4 and 8h with HSV-2 (1 MOI), processed for IFA, reacted with anti-γH2AX antibody followed by Alexa Fluor-594 secondary antibody and DAPI (blue). The boxed areas are enlarged. 40X magnification. **(D)** Total γH2AX foci in nuclei of the cells counted over the entire image as a measurement of amount of γH2AX present in the nuclei.

In uninfected cells, cGAS (red) is present in cytoplasm and can also be seen in the nucleus, while Beclin-1 (green) is seen in the cytoplasm and at low levels in the nucleus (**Figure 6A, B**). Post HSV-2 infection, cGAS increases in the cytoplasm at 2h, 4h and 8h, while Beclin-1 increases both in the cytoplasm and nucleus at 2h, 4h and 8h. Interestingly, cGAS and Beclin1 are seen to interact in the cytoplasm (yellow spots) as well as in the nucleus (white color), in the uninfected condition, as well as at 2h, 4h and 8h post HSV-2 infection. Through the course of infection, their interaction increases in the cytoplasm, as evident by increasing intensity of yellow spots, while their interaction decreases in the nucleus, as evident by the reduced intensity of white color. These observations support the immuno-precipitation results (**Figures 2A**, **3A**).

In **Figure 3B** we found that γH2AX level was increased in the nucleus, similarly, when immunofluorescence assay was performed, in uninfected condition, γH2AX is not observed (**Figure 6C**). Upon HSV-2 infection, it is observed at 4h in the nucleus, which was further enhanced at 8h, in the nucleus, with the puncta becoming more distinct (**Figures 6C, D**). This suggests activation of the DDR pathway at 4h and 8h post HSV-2 infection in HFF cells.

### HSV-2 targets STING signalosome through ICP27

**Figure 7:**
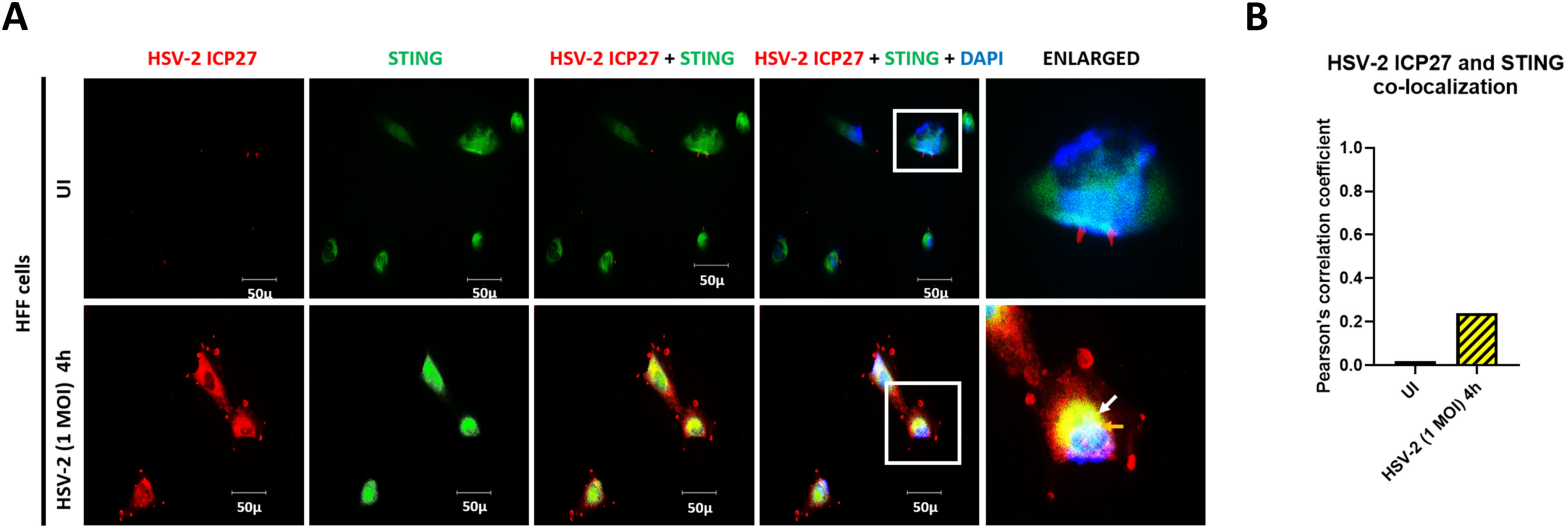
HSV-2 ICP27 targets STING. **(A)** HFF cells were infected for 4h with HSV-2 (1 MOI), processed for IFA, reacted with anti-HSV-2 ICP27 and anti-STING antibodies followed by Alexa Fluor-594/488 secondary antibodies and DAPI (blue). The boxed areas are enlarged. Colocalization of HSV-2 ICP27 with STING at the nuclear membrane and perinclear region (yellow spots, white arrows). 40X magnification. **(B)** Pearson’s correlation coefficient among the red and green channels over the entire field as a measurement of co-localization of HSV-2 ICP27 and STING in the cytoplasm.

In uninfected HFF cells, STING is seen in the cytoplasm (**Figure 7A**). HSV-2 ICP27 protein is absent, and the few red spots seen might be due to non-specific binding of the antibody. After 4h post infection, HSV-2 ICP27 is seen in the cytoplasm, and is highly concentrated in small circular areas in the extremities of the cytoplasm. The amount of STING in the cytoplasm is also substantially increased (**Figures 7A, B**). Importantly, HSV-2 ICP27 and STING are seen to interact in the cytoplasm (yellow spots, white arrows) and around the nucleus (white color at the nuclear periphery). This observed interaction contributes towards the investigations of the role of herpesvirus ICP27 in reduction of IRF3 phosphorylation through targeting of TBK1 activated STING signalosome (69), which is suggested to be dependent on the cell type, and moreover, has not been studied in detail in case of HSV-2.

### HSV-2 infection in HFF cells induces cGAS-independent IFI16 acetylation, inflammasome formation and initial IFI16-STING association for IRF3 activation

**Figure 8:**
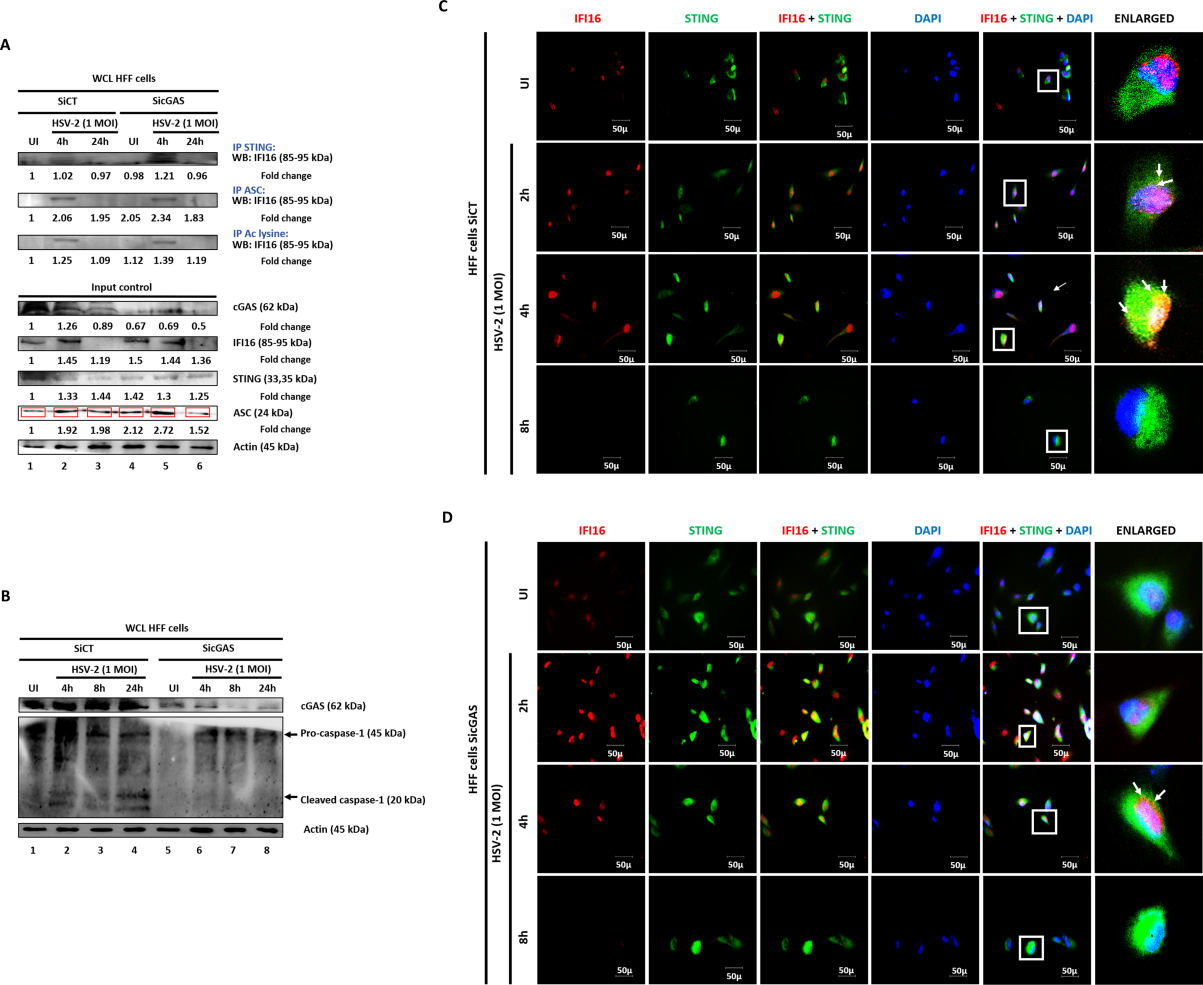
HSV-2 infection in HFF cells induces cGAS-independent IFI16 acetylation, inflammasome formation and initial IFI16-STING association for IRF3 activation. **(A)** HFF cells treated with SiCT or SicGAS were left uninfected or infected with HSV-2 at 1MOI for 4 or 24h. Equal amounts of whole cell lysate proteins were immuno-precipitated with anti-STING, ASC or Acetylated lysine antibody and western blotted for IFI16. Input controls of cGAS, IFI16, STING and ASC are represented, with Actin as loading control. Fold changes are included at the bottom of the respective blots. **(B)** HFF cells treated with SiCT or SicGAS were left uninfected or infected with HSV-2 at 1 MOI for 4, 8 or 24h. whole cell lysate proteins were western blotted for cGAS and cleaved-Caspase-1, with Actin as loading control. **(C)** Mock or **(D)** cGAS knockdown HFF cells were infected for 2, 4 or 8h with HSV-2 (1 MOI), processed for IFA, reacted with anti-IFI16 and anti-STING antibodies followed by Alexa Fluor-594/488 secondary antibodies and DAPI (blue). The boxed areas are enlarged. Co-localization of IFI16 with STING in the cytoplasm (yellow spots). 40X magnification.

KSHV, HSV-1 and EBV infections induce IFI16 acetylation which is responsible for the nuclear to cytoplasmic translocation of IFII6, and interaction of IFI16 with STING, necessary for the activation of IRF-3-dependent IFN-β response (26). Although IFI16 and cGAS interact, the nature of the interaction, its impact on IFN-β response and the role of cGAS in inflammasome function are not known. Immunoprecipitation analysis of SiCT or SicGAS transfected HFF cells, left uninfected or infected with HSV-2, demonstrated the association of IFI16 with STING at 4 h.p.i. which was independent of cGAS, and no association was seen at 24 h.p.i. (**Figure 8A**, upper panel). These results validated that early interaction of STING with IFI16 is cGAS-independent but the 24 h.p.i. interaction was lost as IFI16 was degraded due to HSV-2 infection. Probably, IFI16 alone or in combination with other molecule(s) other than cGAS translocates to the cytoplasm and interacts with STING as shown earlier (26, 27, 36).

IFI16 is associated with ASC for inflammasome formation, and cGAS knockdown did not affect IFI16-ASC association but acetylation of IFI16 was slightly reduced at 4 h.p.i. (**Figure 8A**). These results suggested that at earlier time points up to 4 h.p.i., STING-IFI16 and IFI16-ASC association were not dependent on cGAS. Although we also observed a slight reduction in IFI16 acetylation, when compared with the total protein level, the amount of IFI16 was reduced in the cGAS knockdown cells suggesting that cGAS was not affecting the acetylation of IFI16. A decrease in IFI16 levels corroborates the previous finding of IFI16 stabilization by cGAS in HSV-1 infected cells (33). Efficient knockdown of cGAS and loss of IFI16 by HSV-2 infection are shown in **Figure 8A** input control panel. Levels of STING and ASC are also shown. Actin was used as loading control (**Figure 8A**, input control).

Since we observed the interaction of cGAS with IFI16, NLRP3, ASC as well as pro-Caspase-1 and cleaved-Caspase-1 (**Figures 2A**, **3A**), we questioned whether cGAS plays a positive or negative role for the inflammasome activation. SiCT or SicGAS transfected HFF cells were either left uninfected or infected with HSV-2 and analyzed for cleaved-Caspase-1 (**Figure 8B**). In mock transfected cells, cleaved-Caspase-1 was observed at all time points post infection, however, in cGAS knockdown cells, cleaved-Caspase-1 was absent at all time points post infection, speculating that cGAS is required for the cleavage of Caspase-1 during *de novo* HSV-2 infection. Thus, cGAS positively regulates inflammasome activation. Actin was used as loading control.

Interestingly, although cGAS knockdown does not affect IFI16 acetylation, it inhibits Caspase-1 cleavage (**Figure 8B**), suggesting that the cGAS is in the complex but not facilitating the assembly of IFI16 inflammasome but mediating inflammasome activation by Caspase-1 autocleavage. This finding suggests that only inflammasome assembly is not sufficient for autocleavage of Caspase-1 but other factors are also involved like we are finding cGAS in this experiment.

Mock transfected HFF cells either left uninfected or were infected with HSV-2 for 2h, 4h or 8h, and thereafter processed for IFA with anti-IFI16 and anti-STING antibodies (**Figure 8C**). IFI16 was seen in the nucleus in the uninfected condition at low levels, and after infection, its levels increase at 2h and 4h, in the nucleus and in the cytoplasm, indicating nuclear to cytoplasmic translocation. Thereafter, IFI16 was not observed at 8h, suggesting ICP0-mediated degradation. STING was observed in the cytoplasm in uninfected condition as well as post infection at 2h, 4h and 8h. IFI16 and STING were not seen to interact in uninfected condition. Interaction of IFI16 and STING (yellow color) was seen in the cytoplasm at 2h, which increased at 4h post infection, while interaction was absent at 8h.

cGAS knocked down HFF cells were either left uninfected or infected with HSV-2 for 2h, 4h or 8h and processed for IFA with anti-IFI16 and anti-STING antibodies (**Figure 8D**). The patterns of the presence of IFI16 and STING in the cells in the uninfected condition and upon HSV-2 infection at 2h, 4h and 8h were similar compared the mock transfected cells. Importantly, the interaction of IFI16 and STING (yellow color) was still observed in the cytoplasm of cGAS knockdown cells at 2h and 4h post HSV-2 infection, while not at 8h. However, the intensity of IFI16-STING interaction was slightly lower at these time points, as compared to that in mock transfected cells.

In line with the above observations (**Figure 8A**), these observations indicate that at earlier time points up to 4 h.p.i., the interaction of IFI16 and STING were not dependent on cGAS. However, in the absence of cGAS, this interaction is slightly reduced at 2h and 4h post HSV-2 infection. Indeed, this is reflected by the reduced levels (approximately 2.2-fold) of secreted IFN-β in cGAS knocked-down HFF cells at 4 h.p.i. compared to SiCT cells (**Figure 1B**), again corroborating with the role of cGAS in stabilizing IFI16 during HSV-1 infection (33) and thus, suggestively in the IFI16-STING pathway of IFN-β production at early time points of *de novo* HSV-2 infection in HFF cells.

### Genome pull down demonstrates the association of IFI16 and cGAS with viral genomes in the nucleus

**Figure 9:**
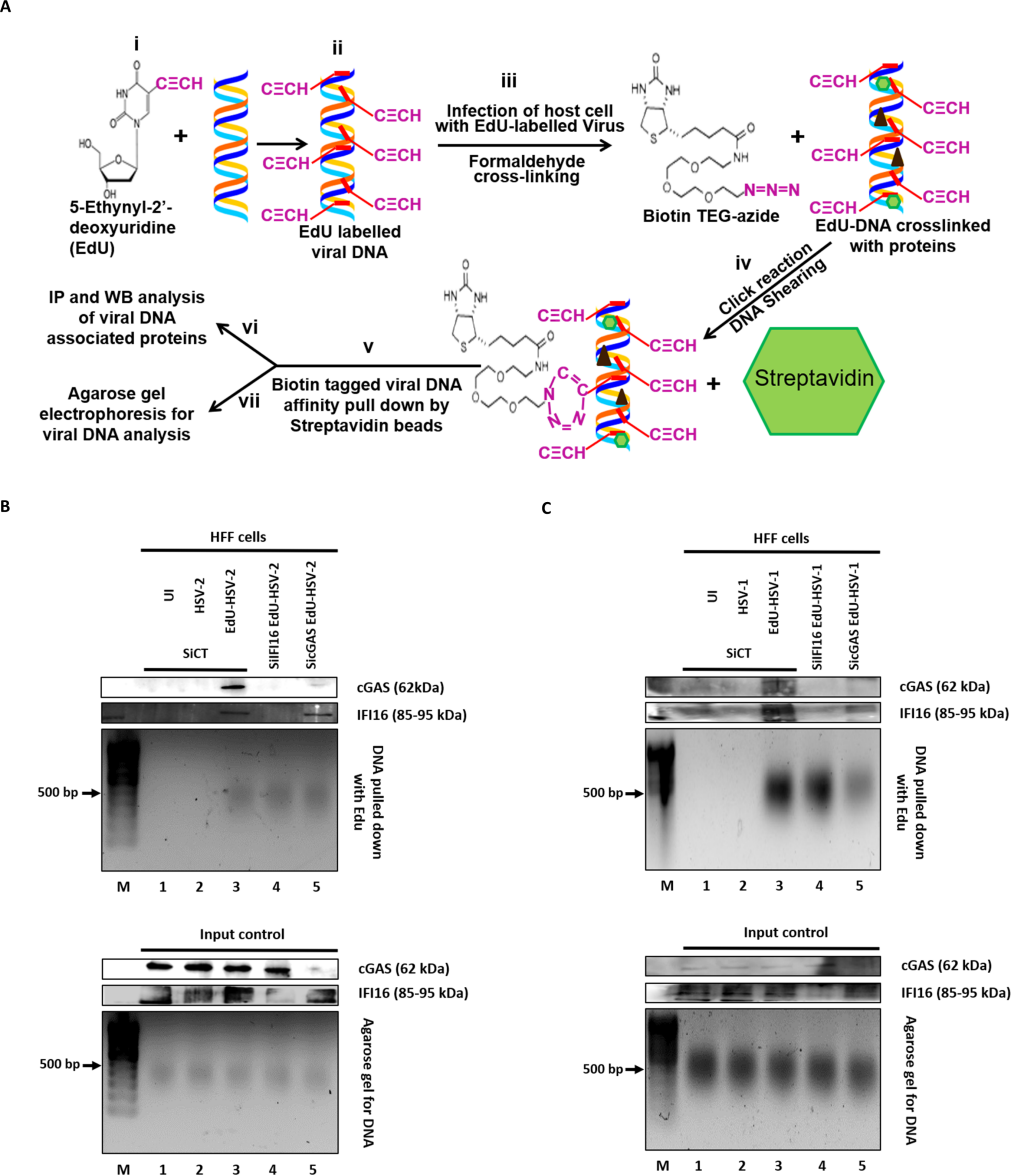
Genome pull down demonstrates the association of IFI16 and cGAS with viral genomes in the nucleus. **(A)** Model depicting the process and major steps involved in the genome pull down assay carried out. After preparation of EdU genome-labelled HSV-2 (i-ii), HFF cells either left uninfected or infected with WT-HSV-2 (10 PFU/cell) or EdU genome-labeled HSV-2 (10 PFU/cell) for 2h (iii), protein-DNA cross-linking was done using formaldehyde. Then biotin-TEG azide was selectively linked to the reactive alkyne group of EdU containing DNA by Click reaction (iv). DNA shearing was performed and the small chromatin fragments captured by streptavidin beads (v). Streptavidin captured samples were used to isolate proteins and DNA. Proteins and DNA from the input materials were analyzed by western blotting (vi) and agarose gel electrophoresis (vii), respectively. HFF cells treated with SiCT, SiIFI16 or SicGAS were infected with WT or EdU-labelled HSV-2 **(B)** or HSV-1 **(C)** for 2h. The proteins were IPed and western blotted. An agarose gel was used to analyze DNA in the samples. Top panels represent the host proteins pulled down with EdU-labelled viral genome. Lower panels show the DNA fragments pulled down with EdU. Input control panels: show the presence of IFI16 and cGAS proteins in the samples. The agarose gel shows the presence of DNA in SiCT, SiIFI16 or SicGAS treated HFF cells.

cGAS recognition of cytoplasmic dsDNA results in the production of cGAMP and activation of the STING-IFN-β response and our studies demonstrate for the first time the association of cGAS with viral genome in the nucleus. To verify this, we used viral genome pull down assays (**Figure 9A**). HFF cells electroporated with SiCT, SiIFI16 or SicGAS were infected with EdU labelled or unlabeled HSV-2 for 1h. Formaldehyde treatment crosslinked the proteins and DNA, then biotin-TEG azide was selectively linked to the viral DNA by the reactive alkyne group of EdU via Click reaction. Pulled down DNA was sheared, fragments captured with streptavidin beads binding to the biotin tagged HSV-2 genome fragments and genome bound proteins. Similar to our earlier findings of IFI16 binding to KSHV and HSV-1 genomes, IFI16 was pulled down with EdU labelled HSV-2 genome and cGAS was also pulled down (**Figure 9B**). Similar amounts of IFI16 and cGAS were detected in the input control and DNA was pulled down only in the EdU group showing the Click reaction specificity. When IFI16 was knocked down, cGAS was not associated with viral DNA, although it was detected in the input control. These results substantiated the **Figure 1B** findings that in the absence of IFI16 the initial IFN-β response was completely abolished. Even in the absence of cGAS, the association of IFI16 with viral genome was not affected, which confirmed that cGAS is not involved in the ability of IFI16 to recognize the viral genome in the nucleus. Although similar amounts of DNA were detected in the input control of uninfected, unlabeled HSV-2 and EdU-HSV-2 infected HFF cells, only EdU labelled genome was pulled down in SiCT, SiIFI16 and SicGAS groups. These results demonstrated that cGAS is not required for IFI16’s viral genome recognition function and IFI16 is required for cGAS’s association with the viral genomes in the nucleus. Since this is the very first report that cGAS is associated with viral genomes in the nucleus, we sought to investigate whether cGAS also interacts with HSV-1 genome in the nucleus. cGAS was pulled down in SiCT but not in SiIFI16 or SicGAS EdU genome labelled HSV-1 infected cells. In contrast, IFI16 was pulled down in SiCT and SicGAS cells and not detected in SiIFI16 cells (**Figure 9C**). These results demonstrated that although both IFI16 and cGAS are associated with HSV-2 genome in the nucleus, IFI16’s association with genome is independent of cGAS, and association of cGAS with nuclear herpes viral genomes requires IFI16.

### IFI16 is required for the association of cGAS with viral genome in the nucleus, whereas cGAS is not required for the nuclear viral genome recognition by IFI16

**Figure 10:**
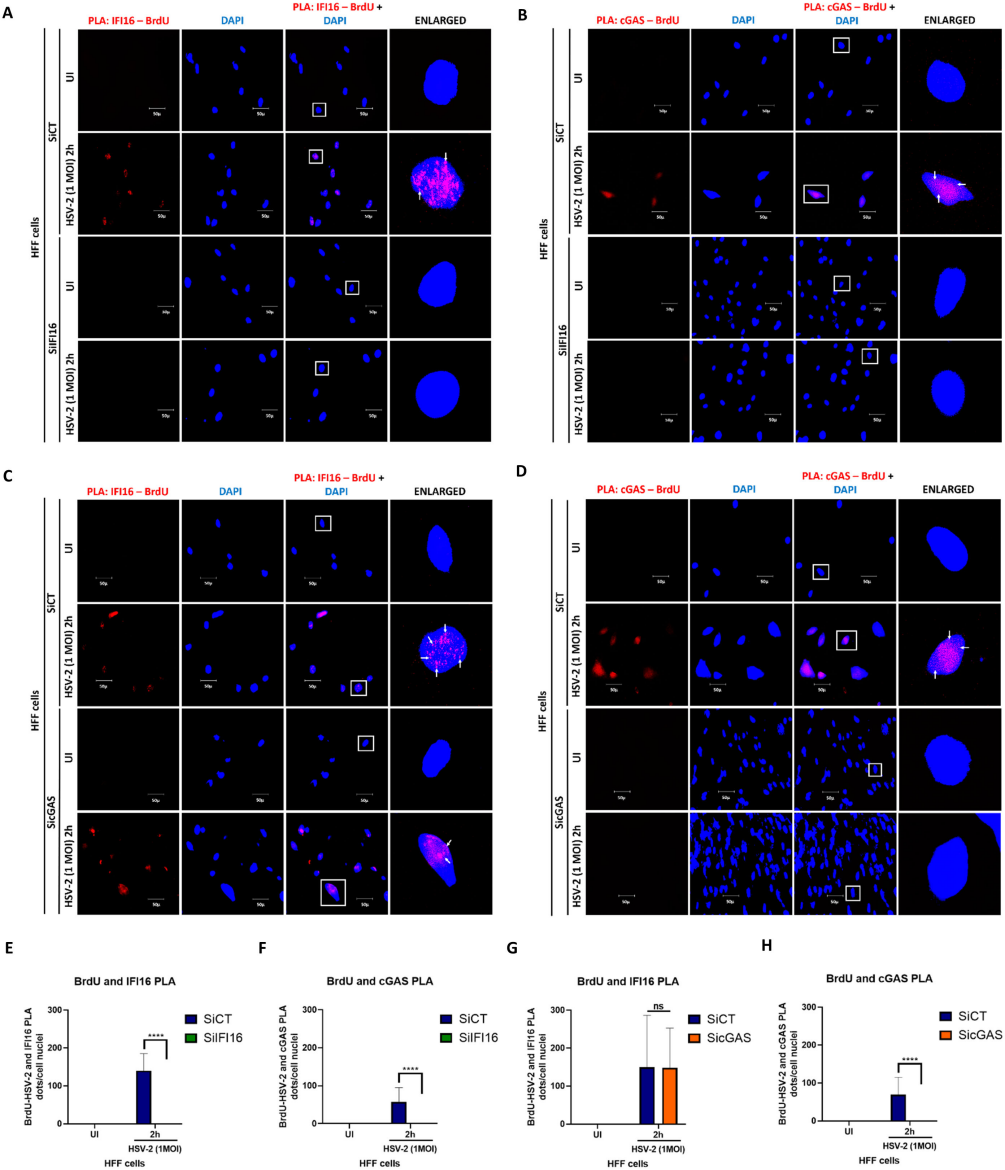
IFI16 is essential for the association of cGAS with the viral genome in the nucleus but cGAS is dispensable for IFI16-mediated genome recognition. **(A, B)** HFF cells electroporated with SiCT or SiIFI16 were left uninfected or infected with BrdU genome-labelled HSV-2 (1 MOI) for 2h and associations of IFI16 **(A)** or cGAS **(B)** with the BrdU - labelled HSV-2 genome were analyzed by PLA using anti-IFI16 or anti-cGAS and anti-BrdU antibodies. Red dots (white arrows): IFI16-BrdU-HSV-2 **(A)** or cGAS-BrdU-HSV-2 **(B)** association in the nuclei. DAPI (blue): nuclei. **(C, D)** HFF cells electroporated with SiCT or SicGAS were left uninfected or infected with BrdU - genome labelled HSV-2 (1 MOI) for 2h and associations of IFI16 **(C)** or cGAS **(D)** with the BrdU - labelled HSV-2 genome were analyzed by PLA using anti-IFI16 or anti-cGAS and anti-BrdU antibodies. Red dots (white arrows): IFI16-BrdU-HSV-2 **(A)** or cGAS-BrdU-HSV-2 **(B)** association in the nuclei. DAPI (blue): nuclei. **(E–H)** Average PLA dots/cell in SiCT and SiIFI16 or SicGAS transfected and HSV-2 infected HFF cells. The data represent the mean of three determinants ± S.D. ****p<0.0001. NS, non-significant.

Mock transfected or IFI16 knocked down HFF cells were either left uninfected or infected with BrdU genome-labelled HSV-2 for 2h and processed for PLA with anti-IFI16 and anti-BrdU antibodies. In mock transfected and uninfected cells, no PLA dots were observed. After 2h of infection in the mock transfected cells, distinct PLA puncta were observed in the nucleus (**Figure 10A**, red dots, white arrows and **Figure 10E**), representing the interaction of IFI16 with the BrdU-labelled genome of HSV-2 present in the nucleus, confirming that nuclear IFI16 senses the HSV-2 genome. PLA dots were not observed in IFI16 knocked down HFF cells in uninfected as well as infected condition, further validating the specificity of the interaction.

Mock transfected or IFI16 knocked down HFF cells were either left uninfected or infected with BrdU genome-labelled HSV-2 for 2h and processed for PLA with anti-cGAS and anti-BrdU antibodies. In mock transfected and uninfected cells, no PLA dots were observed. After 2h of infection, PLA dots, although diffused, were observed in the nucleus, representing interaction of cGAS with the BrdU-labelled genome of HSV-2 present in the nucleus (**Figure 10B**, red dots, white arrows and **Figure 10F**). Although cGAS was associated with BrdU-labelled genome in SiCT, the number of cGAS molecules associated with genome was about 3 times less than those of IFI16 molecules in the SiCT cells (**Figure 10E, F**). Further, in IFI16 knocked down and uninfected cells, no PLA dots were observed, validating the specificity of the interaction. However, interestingly, no PLA dots were observed in IFI16 knocked down and infected cells at 2h, indicating that cGAS does not interact with the BrdU-labelled genome of HSV-2 in the absence of IFI16.

Together, these observations indicate that early during infection, cGAS does not directly and independently interact with the nuclear input HSV-2 genome, and its interaction is dependent on the presence and interaction of IFI16 with the nuclear HSV-2 genome. The observed increase in IFI16-viral genome PLA spots in the infected cells probably reflects the multiple roles played by IFI16 during HSV-1 and KSHV infection such as the viral gene transcription regulation, epigenetic modulation, and inflammasome and interferon responses. It is possible that cGAS association with viral genome may be mostly involved in the IFN-β response as we are not aware of any other function of cGAS in the nucleus.

Mock transfected or cGAS knocked down HFF cells were either left uninfected or infected with BrdU genome-labelled HSV-2 for 2h and processed for PLA with anti-IFI16 and anti-BrdU antibodies. In mock transfected and uninfected cells, no PLA dots were observed. After 2h of infection in the mock transfected cells, distinct PLA puncta were observed in the nucleus, representing the interaction of IFI16 with the BrdU-labelled genome of HSV-2 present in the nucleus, confirming that nuclear IFI16 senses the HSV-2 genome (**Figure 10C**, red dots, white arrows and **Figure 10G**). Further, no PLA dots were observed in cGAS knocked down uninfected cells, validating the specificity of the interaction. However, distinct PLA dots were observed in the cGAS knocked down cells after 2h of infection (**Figure 10G**), indicating that IFI16 can interact with the BrdU-labelled genome of HSV-2 in the nucleus even in the absence of cGAS. Moreover, the PLA dots observed in cGAS knocked down cells were lower in number than that in mock transfected cells, indicating that in the absence of cGAS, interaction of IFI16 with the BrdU-labelled genome of HSV-2 in the nucleus is hindered.

Mock transfected or cGAS knocked down HFF cells were either left uninfected or infected with BrdU genome-labelled HSV-2 for 2h and processed for PLA with anti-cGAS and anti-BrdU antibodies. In mock transfected and uninfected cells, no PLA dots were observed. After 2h of infection in the mock transfected cells, distinct PLA puncta were observed in the nucleus, representing the interaction of cGAS with the BrdU-labelled genome of HSV-2 present in the nucleus (**Figures 10D**, red dots, white arrows and **Figure 10H**). PLA dots were not observed in cGAS knocked down HFF cells in uninfected as well as infected condition, further validating the specificity of the interaction.

Together, these observations indicate that IFI16 can directly interact with the nuclear HSV-2 genome, even in the absence of cGAS, however, optimal interaction is still dependent on the presence of cGAS.

The PLA results indicate that at 2h post HSV-2 infection in HFF cells, IFI16 directly senses the HSV-2 genome and that this sensing occurs only in the nucleus. Further, nuclear cGAS interacts with the HSV-2 genome, but only when IFI16 is already bound to the HSV-2 genome. Furthermore, cGAS is essential for optimal recognition of HSV-2 genome by IFI16. Therefore, in the nucleus, the HSV-2 genome is primarily sensed by IFI16 and cGAS assists IFI16 for optimal sensing, supporting the previously reported role of cGAS in stabilizing IFI16 for viral genome recognition.

### cGAS induces cGAMP production in the nucleus of HFF cells upon *de novo* HSV-2 infection

**Figure 11:**
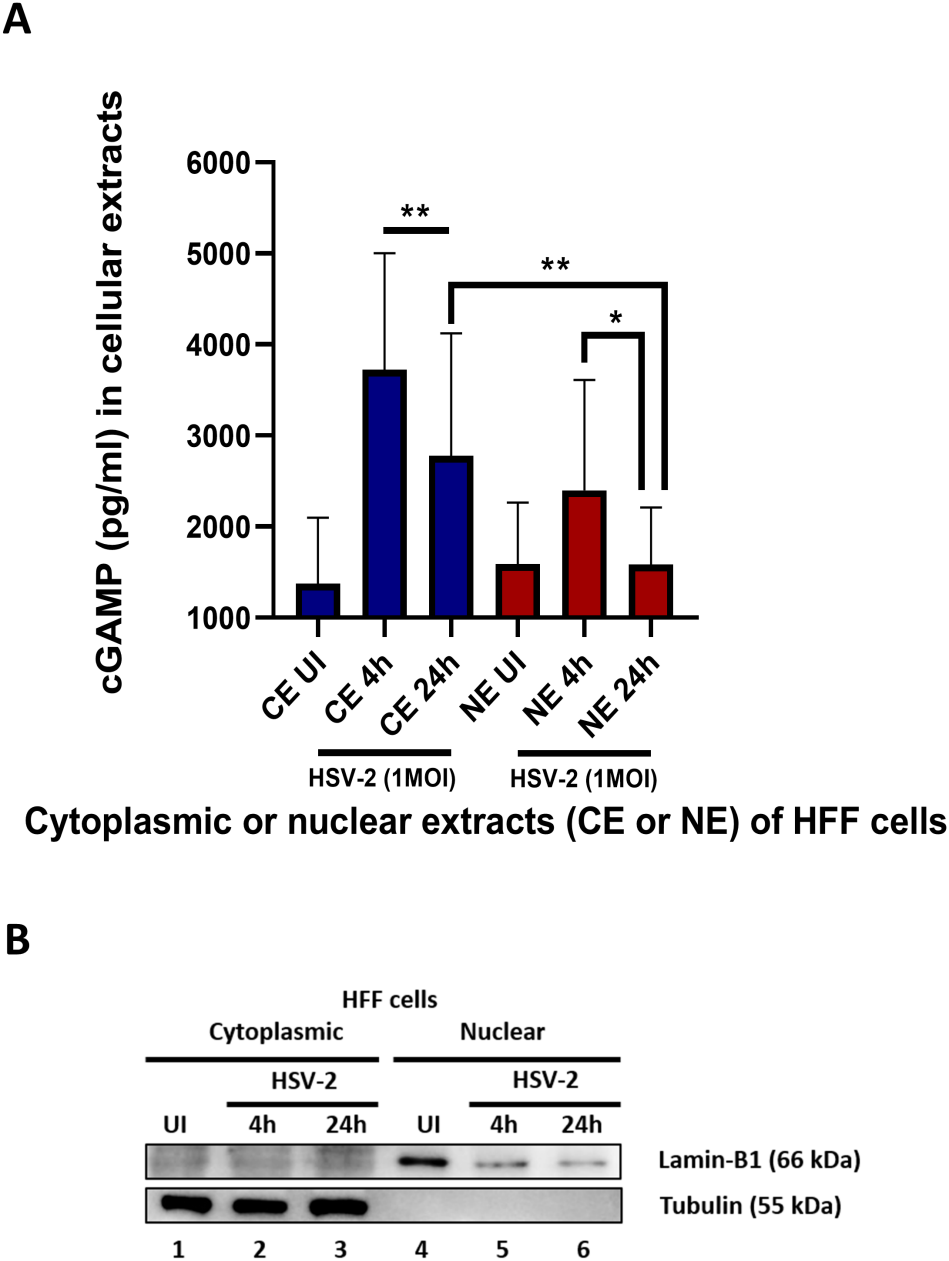
cGAMP levels in the cytoplasmic and nuclear fractions of HFF cells uninfected or infected with HSV-2 at 1 MOI for 4 or 24h were quantified by ELISA. The data represent the mean of three determinants ± S.D. and are representative of three independent experiments with similar observation. *p<0.05, **p<0.01. NS, non-significant. Equal amounts of cytoplasmic and nuclear fractions were western blotted for Lamin-B1 and Tubulin to validate the purity of both fractions.

cGAS recognizes dsDNA in the cytoplasm and produces cGAMP to activate the STING-TBK1-pIRF3-IFN-β response. During *de novo* HSV-2 infection in HFF cells, our studies demonstrate the IFI16-dependent association of nuclear cGAS with incoming viral DNA, and the IFI16-independent association of cGAS with the newly synthesized viral genome in the cytoplasm. This prompted us to investigate whether cGAS could also be enzymatically active in the nucleus, in addition to the cytoplasm. The cytoplasmic and nuclear fractions prepared from HFF cells uninfected or infected with HSV-2 for 4 hours or 24 hours were used for quantifying the levels of cGAMP through ELISA (**Figure 11A**). The purity of the cytoplasmic and nuclear fractions was checked through immunoblotting for Tubulin and Lamin-B1 respectively (**Figure 11B**). Interestingly, cGAMP was detected in the cytoplasmic as well as the nuclear fractions at all time points (**Figure 11A**). cGAMP levels were significantly elevated at 4 h.p.i. in the cytoplasm, while in the nucleus, the levels were slightly increased at 4 h.p.i. At 24 h.pi. the cGAMP levels were reduced both in the cytoplasm as well as the nucleus, however, this reduction was more prominent in the nucleus than in the cytoplasm.

## Discussion

**Figure 12:**
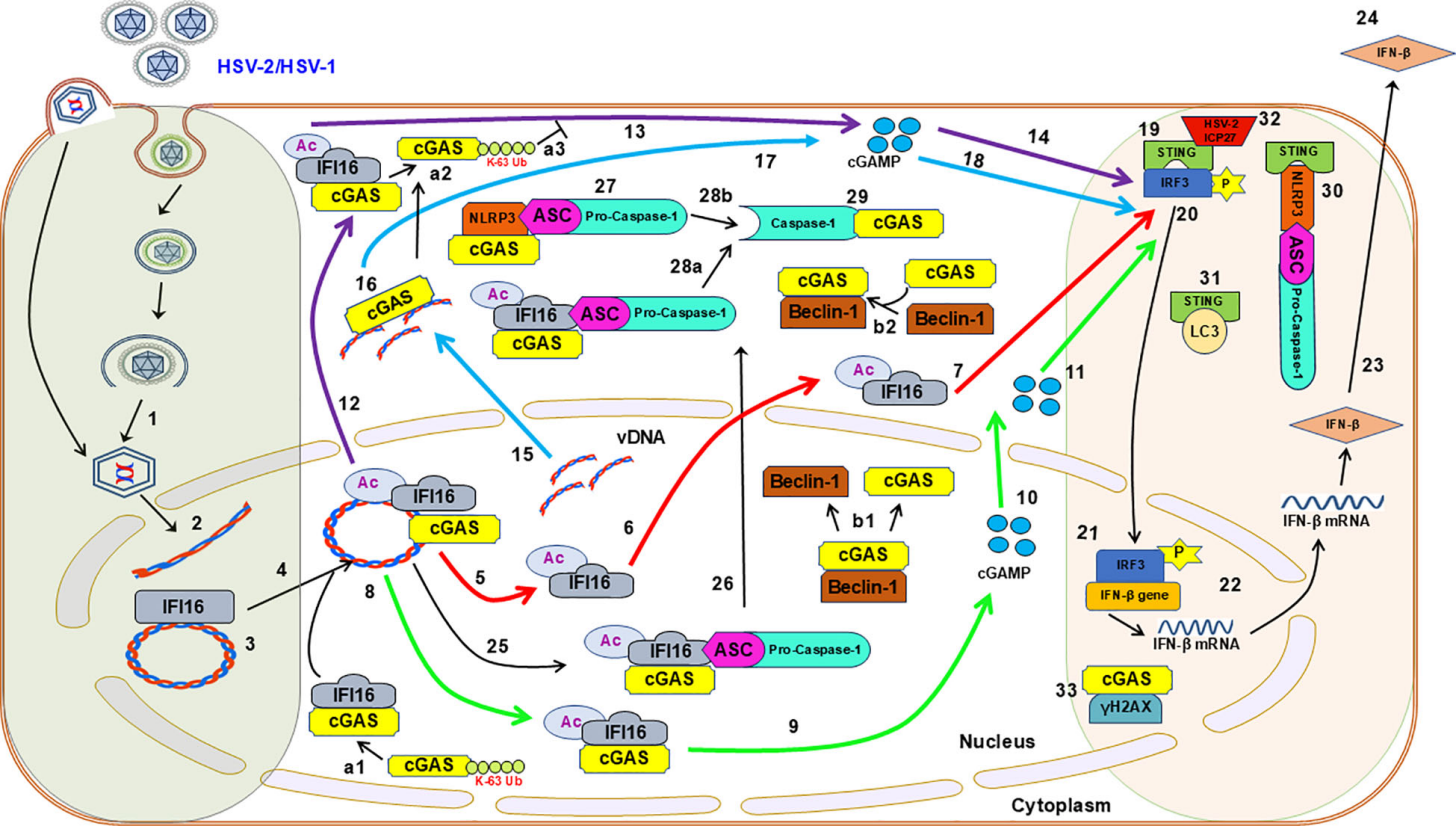
Infographic: (1–3 black lines) Viral DNA entry into the nucleus and recognition by IFI16 and cGAS. (4–7 red lines) Acetylated IFI16 translocates to the cytoplasm and associates with STING and activates it. (8–11 green lines) cGAS is associated with IFI16, and cGASIFI16 complex induces cGAMP in the nucleus which translocates to the cytoplasm and activates STING. (12–14 violet lines) Genome recognition by IFI16 and cGAS leads to translocation of the cGAS-IFI16 complex to the cytoplasm, where it produces cGAMP. (15–18 blue lines) The newly synthesized viral genomic DNA leaks or translocates to the cytoplasm and is then recognized by the cytoplasmic cGAS independent of IFI16, which produces cGAMP. (19–24 black lines) STING activated by any of the discussed pathways activates IRF3 by phosphorylation. The pIRF3 moves to the nucleus leading into the IFN-β gene transcription, cytoplasmic translocation and translation and secretion of IFN-β into the culture supernatant. (a1-a2-a3 black lines) Reduced inhibition of nuclear cGAS and increased inhibition of cytoplasmic cGAS - Reduction in K-63 linked poly-ubiquitination of nuclear cGAS and its simultaneous increase in the cytoplasm. (b1-b2 black lines) Loss of cGAS-Beclin-1 interaction in the nucleus and its simultaneous increase in the cytoplasm. (25–26 black lines) cGAS suggested to be in complex with IFI16 inflammasome in the nucleus and the cytoplasm (27). Simultaneously, cGAS is also suggested to be in complex with the NLRP3 inflammasome in the cytoplasm. (28a-29) Active Caspase-1 interacts with cGAS in the cytoplasm (30). Activated STING suggested to induce the NLRP3 inflammasome (31). STING also interacts with LC3 (32). HSV-2 ICP27 binds to STING. (33). cGAS interacts with γH2AX in the nucleus.

Earlier we have shown that IFI16 recognizes herpes viral genome in the nucleus and instigates inflammasome and type I IFN responses. Furthermore, the co-ordination between IFI16 and cGAS in the nucleus has also been reported, however, the nature of co-ordination remains unclear. Herein, we do not only reveal for the first time that IFI16 recognizes incoming HSV-2 DNA in the nucleus, independent of cGAS, but also reveal that nuclear viral genome recognition by cGAS is IFI16-dependent (**Figures 8**–**12**). In addition, we have explored 4 different possible pathways of IFN-β production. a) the nuclear cGAS produces cGAMP, which translocates to the cytoplasm, b) the cGAS-IFI16 complex translocates to the cytoplasm and produces cGAMP, c) the acetylated IFI16 translocates to the cytoplasm and directly interacts with STING, and d) the newly synthesized viral genome is recognized by cytoplasmic cGAS which produces cGAMP. All pathways lead to production of IFN-β through STING-TBK1-IRF3 axis. Thus, at early time point of infection, the IFN-β production is mediated by both, the nuclear cGAS as well as IFI16, through STING activation. Moreover, IFI16 acetylation (**Figures 2B, C**, **8A**) also results in interaction of IFI16 with ASC and assembly of inflammasome, increased interaction with Ran-GTPase and cytoplasmic redistribution, and activation of Caspase-1, resulting in IL-1β production, as described earlier (26).

The IFI16 and cGAS interaction leads to biphasic production of type I IFN response (**Figure 1B**). This coordination remains unique in the sense that cGAS is involved in both nuclear as well as cytoplasmic viral DNA sensing and promotes stability of IFI16-viral gnome interaction in the nucleus, on the other hand, the IFI16 mediates cGAS interaction with viral genome in the nucleus (**Figure 12**).

The acetylation of IFI16 facilitate its nuclear to cytoplasmic translocation where it binds to STING to activate the TBK1-IRF3 axis. IFI16 acetylation is also essential for its interaction with ASC and inflammasome assembly in the nucleus as well as its cytoplasmic translocation and activation (**Figure 12**, Red lines). It is also possible that IFI16 translocates to the cytoplasm either alone or in complex with molecules other than cGAS, such as histone H2B or BRCA-1 (26, 27, 36). Infection induced IFI16 acetylation was not dependent on cGAS (**Figures 8A, C, D**).

The interaction of cGAS with IFI16, ASC and Caspase-1 in the nucleus and the cytoplasm (**Figure 3A**), cGAS-independent IFI16 acetylation (**Figure 8A**) and Caspase-1 cleavage-inhibition upon cGAS knockdown (**Figure 8B**) suggest that cGAS plausibly aids the Caspase-1 cleavage in IFI16 inflammasome complex in the nucleus. Additionally, similar suggestion could be made for NLRP3-mediated Caspase-1 cleavage in the cytoplasm (**Figures 2A**, **8B**).

Interestingly, we also observed the interaction of cGAS with STING in HFF cells (**Figures 2B, C**). The low amount of cGAS-STING interaction observed in uninfected condition, which consistently increased at 4 hours and 8 hours post infection, thereafter reduced at 24 hours, raises important questions, such as which domains of the two proteins are involved in this interaction, whether cGAS bound to STING is also bound to viral DNA in the cytoplasm, whether it is enzymatically active, how interaction with cGAS affects the activity of STING. Further investigations to answer these questions could lead to elucidation of another pathway operating in innate immunity. The importance of cGAS-STING interaction being maximal at 8 hours post HSV-2 infection in HFF cells, in the context of other mechanisms operating at this time point, needs to be understood.

The activation of inflammasome response during HSV-2 infection in HFF cells (**Figure 2D**) was evidenced by cleaved-Caspase-1, cleaved-IL-1β and cleaved-IL-18, which were found to be elevated at 4 hours and 8 hours post infection and reduced at 24 hours, while IL-18 still increased at 24 hours. Earlier, HSV-1 infection in HFF cells has been shown to induce activation of the IFI16 and NLRP3 inflammasomes early during infection (studied at 2 hours and 4 hours), and subsequent selective degradation of IFI16 and suppression of the NLRP3 inflammasome. The HSV-1 tegument protein VP22 was shown to interact with AIM2 and prevent its oligomerization (70). Similar mechanisms may be operational during HSV-2 infection in HFF cells. Importantly, increase in cleaved-Gasdermin-D levels at 4 hours and 8 hours post HSV-2 infection suggest increased transient pyroptosis in HFF cells.

The cGAS-STING-NLRP3 complex activates the NLRP3 inflammasome through two approaches – (i) improves ER to Golgi translocation of STING (ii) STING and NLRP3 interaction attenuate K48- and K-63-linked polyubiquitination of NLRP3, both events lead to NLRP3 inflammasome activation (71–74). Interestingly, reduced STING-NLRP3 interaction at late time points post HSV-2 infection in HFF cells suggest reduced NLRP3 inflammasome (**Figure 2B**). Previous studies involving HSV-1 infection or cytosolic DNA stimulation in THP1, HEK293T, Hela cells, as well as in mice Bone-Marrow-Derived macrophages (BMDMs) and primary mouse embryo fibroblasts (MEFs) cells have demonstrated the similar results. Ours is the first report of STING-NLRP3 interaction during HSV-2 infection in HFF cells. Thus, inflammasome activation at early time point (4 hours post infection) seems to be mediated by both IFI16 and STING-NLRP3 axis. At later time points, IFI16 is degraded, and inflammasome response seems to be dependent on NLRP3, by STING and/or other upstream stimuli. STING and ASC interaction was also observed at early time point post infection, which thereafter reduced at later time point, similar to the STING-NLRP3 interaction pattern.

HSV-1 induced active Caspase-1, directly interacts with cGAS, cleaves at D^140/157^ in the N-terminal results into reduced cGAMP, hence, hampered cGAS–STING signaling. Thus, suggesting a cross-regulation between the type I IFN and inflammasome pathways induced by intracellular DNA sensors (42). During HSV-2 infection in HFF cells, we observed the cGAS-pro-Caspase-1 interaction even in uninfected condition which increases maximally during early infection time as seen in whole cell lysate. In the uninfected cells while the IFI16 and cGAS interact with each other in the nucleus, interaction increases during HSV-2 infection, and the cGAS interacts with IFI16, ASC and pro-Caspase-1, probably helping in inflammasome activation (**Figure 3A**). Whereas, the cGAS-cleaved-Caspase-1 interaction was detected at 4h post infection, reduced at 8h, and surprisingly the interaction was enhanced at 24h, possibly, involved in cGAS cleavage (**Figure 2A**). This phenomenon looks like regulation of both inflammasome and type I IFN response. Hence, deeper investigations are warranted in terms of the mechanism and magnitude of its effect on IFN-β production and viral clearance or evasion.

*De novo* HSV-2 infection in HFF cells induces autophagy and DDR pathways which are involved in intricate crosstalk with type I interferon pathway and mutual regulation. Elevated expression levels of Beclin-1, ATG5, ATG5-ATG12 complex as well as LC3 I and LC3 II collectively indicate that *de novo* HSV-2 infection in HFF cells induces autophagy, which approaches towards the maturation stage at later time point post infection. Importantly, Beclin-1 was present in the nucleus (**Figures 3B**, **6A**) where it was increased during infection at 4 and 24 hours, and moreover, the levels in the nucleus were slightly higher than that in the cytoplasm at later time points (**Figure 3B**). Previously, Beclin-1 isoforms formed due to alternative splicing have been reported to have different effects on autophagy. Indeed, in addition to the typical 60 kDa band of Beclin-1, we also observed the presence of bands of different molecular weights in the WCL and cytoplasmic and nuclear fractions (**Figures 2D**, **3B**). Also, their distribution in the cytoplasm and nucleus differed (**Figure 3B**). It will be interesting to further investigate the presence and role of the Beclin-1 isoforms in autophagy and apoptosis during HSV-2 infection in HFF cells, and the regulation of these processes.

Importantly, cGAS was found to interact with Beclin-1 during HSV-2 infection in HFF cells (**Figures 2A**, **3A**, **6A, B**). In whole cell lysates, prominent cGAS-Beclin-1 interaction at 4 and at 8 hours post infection was observed. Further, their interaction was observed to decrease in the nucleus and increase in the cytoplasm from uninfected to early (4 hours) and late (24 hours) time points post infection. Previously, HSV-1 infection in other cell lines was shown to lead to the direct interaction of cGAS and Beclin-1, resulting in suppression of the cGAS NTase activity, cGAMP synthesis and IFN production, suggesting that cGAS may shuttle between the STING-mediated IFN pathway and the Beclin-1-mediated autophagy pathway, in order to induce IFN production while inducing autophagy-mediated DNA degradation, thus avoiding persistent immune stimulation. Our observations, in addition to the above report, suggest towards a possibility that negative regulation of cGAS by Beclin-1 reduces in the nucleus and increases in the cytoplasm from early through later time points post HSV-2 infection in HFF cells. These dynamic interactions suggest towards a very interesting and possibly efficient regulation of nuclear as well as cytoplasmic cGAS activity and innate immune function during HSV-2 infection in HFF cells, highlighting a crucial role of Beclin-1 in the nucleus.

Significant Beclin-1-cGAS interaction in uninfected condition observed here, might be important for suppressing its DNA sensing ability and thus enzymatic activity, preventing possible aberrant sensing of host chromosomal DNA by cGAS molecules that are nuclear soluble and not bound to the H2A-H2B of nucleosomes (which keep cGAS incapable of DNA sensing). Upon *de novo* HSV-2 infection, incoming HSV-2 genomic DNA enter the nucleus, reportedly inducing the cGAS molecules to become free from the nucleosomes and nuclear soluble. Further, as we report here, at early time point, nuclear IFI16 senses the HSV-2 genome, which allows nuclear cGAS to bind to the viral genome. The reduced interaction of nuclear cGAS and Beclin-1 observed at early time point could be correlated to the above observation, that a part of cGAS is capable of and involved in viral DNA binding (dependent on IFI16) and is not suppressed by Beclin-1. This could also explain the low levels of cGAS enzymatic activity for cGAMP production in the nucleus that we observed. At later time point (24 hours), the cGAS-Beclin-1 interaction in the nucleus further reduces, probably since IFI16-mediated viral genome sensing in the nucleus does not occur, cGAS DNA-sensing and enzymatic activity in the nucleus also do not occur, and therefore, do not need high level of suppression. While in the cytoplasm, in uninfected condition, the low levels of cGAS-Beclin-1 interaction suggests a strategy of the host to keep regulatory check on cGAS sensing of aberrant DNA, such as mtDNA leaking into the cytoplasm, or chromosomal DNA fragments or micronuclei due to mitosis. At early time point of infection, since the cytoplasm does not contain much newly synthesized viral DNA, a suppressive regulation would not be required in much higher amount, indicated by only a slight increase in cGAS-Beclin-1 interaction at 4 hours. While in the cytoplasm, at later time point, we report that cGAS senses the newly synthesized viral DNA independent of IFI16 and synthesizes cGAMP leading to IFN-β. Here, the observed increased levels of cGAS-Beclin-1 interaction suggest that a high level of negative regulation of at least a fraction of cytoplasmic cGAS could either be required (beneficial for the host to avoid over activation) or induced by the virus (for immune evasion). It would also be important to investigate whether other DNA sensors, such as IFI16, DDX41 also interact with Beclin-1 and induce autophagy.

Importantly, we also found that upon *de novo* HSV-2 infection in HFF cells, STING interacts with LC3, only at 8h post infection (**Figures 2B, C**). Earlier, it has been reported that upon dsDNA stimulation, STING interacts with ATG9a and LC3 leading to autophagy (75), and upon HSV-1 infection, LC3 punctum are formed and LC3-I is converted to LC3-II (42). This process was inhibited in STING knockout cell lines, highlighting the role of STING in autophagy induction (42, 76). Although the mechanism is not completely understood, STING was reported to also induce autophagy during its translocation, wherein STING-containing ERGIC vesicles are hypothesized to be able to deviate from the IFN production pathway to activate LC3 lipidation through a WIPI2 and ATG5-dependent mechanisms independent of ULK and the VPS34- kinase complex (42, 76, 77).

The DNA damage response induced during *de novo* HSV-2 infection in HFF cells is evident by increased level of ATM and its phosphorylation (**Figure 3B**), and phosphorylated H2AX (γH2AX) puncta, in the nucleus (**Figures 6C, D**). Several components such as ATM, MRN and WRN were reportedly required for efficient virus production (41, 78, 79). Interestingly, the nuclear cGAS was found to be interacting with the γH2AX in the nucleus at 24 hours post HSV-2 infection (**Figure 3A**), correlating with a previous report of this interaction, where DDR induced by etoposide, a genotoxic agent, was responsible for this interaction, which facilitated the recruitment of cGAS to DNA-damage sites. DNA damage leads to cGAS dephosphorylation, inducing its nuclear translocation through the Nuclear Localization Signal2 (NLS-2), independent of DNA sensing function of cGAS (63).

Many herpesviruses encode several lytic proteins and utilize several different mechanisms to block the detection of newly replicated DNA leaked in to the cytoplasm by cGAS. UL37, a tegument protein of HSV-1, deamidates cGAS, disrupting its catalytic function to inhibit the synthesis of cGAMP (80). Recently, identified protein VP24 of HSV-1 disrupts the interactions between TBK1 and IRF3, impairing IRF3 activation and activation of IFN-β promotor (81) HSV-1 VP22 interacts with cGAS, inhibiting its enzymatic activity (82). Another protein with deubiquitinase activity, HSV-1 ubiquitin-specific protease (UL36USP), is thought to primarily target cGAS and STING (83). The γ_1_34.5 of HSV-1 directly interacts with STING, disrupting its transport from the ER to Golgi, to inhibit the activation of TBK1 (84). The ICP27 of HSV-1 is expressed *de novo* in human macrophages, with early nuclear localization followed by later translocation to the cytoplasm, where it interacts with TBK1 and STING in a manner that was dependent on TBK1 activity and the RGG motif in ICP27. This prevented IRF3 activation and type I IFN expression. ICP27 has been reported to be highly conserved among herpesviruses (69). Importantly, HSV-2 ICP27 interaction with STING at early time point post infection in the (**Figure 7A, B**) cytoplasm as well as around the nucleus suggest that this mechanism might be used by the virus early during infection (at 4h) in order to reduce type I IFN response so that it is not too heightened.

The discriminative DNA sensing by cGAS i.e. IFI16-dependent sensing of incoming viral genome in the nucleus and IFI16-independent sensing of newly synthesized viral DNA in the cytoplasm, raises a very interesting question like why cGAS does not sense viral DNA independently in the nucleus but it does so in the cytoplasm. It is established that cGAS affinity for DNA varies from relatively weak (Kd ~ 20 µM) to strong (Kd ~ 80 nM) (30) attributed to various post-translational modifications, the plausible cause for discriminative sensing by cGAS. It seems, the cGAS nuclear function is probably tightly regulated through IFI16 or other unknown factors to avoid innate responses against the host self-nucleic acids. In contrast, where self-DNA act as DAMP in the cytoplasm requires highly sensitive DNA sensors to avoid unwanted DNA accumulation. There must be additional critical pathways to regulate cGAS activation by foreign DNA while being unresponsive to self-DNA in the nucleus. Our studies show that IFI16 is required for DNA sensing and enzymatic activity of nuclear cGAS (**Figures 1**, **9**, **10** and **12**). The cGAS needs the polyglutamine binding protein 1 (PQBP1) as a cofactor to achieve optimal binding to reverse-transcribed HIV-1 DNA in the cytoplasm (85). β-arrestin 2, a multifunctional adaptor, reportedly promotes the recognition of dsDNA by cGAS in macrophages, promoting IFN-β production and clearance of HSV-1 and vesicular stomatitis virus (VSV). However, the detailed mechanism, the residues of cGAS involved and whether regulation is direct or via other enzymes recruited by β-arrestin 2, was not clear (86). ZCCHC3, a CCHC-type zinc-finger protein, acts as a general co-sensor of cGAS, promoting its binding to dis-located self or microbial dsDNA and its enzymatic activity in the cytoplasm. However, it was not known whether individual binding of ZCCHC3 or cGAS to dsDNA promotes the interaction of the two proteins, and subsequently increases the affinity of the complex to dsDNA, or alternatively, viral infection firstly induces formation of ZCCHC3 and cGAS complex through unknown mechanisms, shown to have much higher affinity to dsDNA than each of them alone. ZCCHC3 was reported to be ubiquitously expressed in THP1, HFF, HCT116, A549, HepG2 and HeLa cells, as well as human macrophages and DCs, with expression levels correlating to those of cGAS in all of those except HCT116. Importantly, this study was confined only to the cytoplasmic sensing of dsDNA by cGAS, and HSV-1 infection studies were not conducted in HFF cells (87). It is possible that other different nuclear and cytoplasmic cofactors may be the source of the discriminative sensing pattern of cGAS. Further detailed study is required to define the discriminative sensing by cGAS.

Due to the complex nature of PTMs, the same PTM at different sites of cGAS may result in different outcomes in terms of its activity. AKT regulated Ser305 phosphorylation and TTPPs mediated glutamylation of cGAS inhibit its DNA binding ability and enzymatic activity (29). The carboxylpeptidases CCP5 and CCP6 activate cGAS by reversing the modification. Mitotic kinases ensure that cGAS is inactive when bound to chromatin during mitosis, potentially aiding in prevention of autoimmune responses. Cyclin-dependent kinase 1 (CDK1) cyclin B complex, unlike AKT, phosphorylates mouse cGAS only at S291 and human cGAS at S305 during intermittent mitosis, inhibiting cGAMP synthesis, and upon mitotic exit, protein phosphatase 1 (PP1) reverses this phosphorylation, restoring the DNA-sensing capacity (88). Aurora kinase B-mediated phosphorylation of cGAS N-terminus inhibits oligomerization of chromatin-bound cGAS, hampering cGAS activation, hence blocking chromatin DNA sensing (89). In resting cells, the constitutive association of serine/threonine phosphatase (PSP) PPP6C to cGAS inhibits phosphorylation of mouse cGAS at S420 (human S435), preventing its binding to GTP, thus inhibiting cGAMP synthesis, whereas PPP6C is dissociated upon viral infection (90). Additionally, phosphorylation at Y215 of cGAS mediated by B-lymphoid tyrosine kinase in resting cells controls its cytosolic retention, while dephosphorylation leads to its nuclear translocation (63). Recently, Poly (ADP-ribose) polymerase 1 (PARP1)-mediated PARylation of cGAS in the cytosol was shown to inhibit the DNA-binding ability of cGAS (91). The protein arginine methyltransferase 5 (PRMT5) mediates asymmetric demethylation of cGAS, weakening the anti-viral response (92). Upon binding of DNA to cGAS, palmitoyl S-acyltransferase ZDHHC18 palmitoylates cGAS, inhibiting immune signaling (93). In uninfected cells or during early antiviral immune response, TRIM38 targets cGAS for SUMOylation, preventing K48-linked ubiquitination and degradation (94). In the later stage, sentrin-specific protease 2 (SENP2)-mediated de-SUMOylation of cGAS avoids excessive and potentially harmful immune responses (95). SUMOylation at K335, K372, and K382 of cGAS impair cGAS-DNA binding, oligomerization and enzymatic activity, weakening the antiviral response. Contrarily, SENP7 de-SUMOylates cGAS, relieving this inhibition (96). Addition of NEDD8 (Neddylation) to the lysine residue of cGAS by the Ube2m-RNF111 axis has been suggested to positively regulate DNA sensing by cGAS, however, the mechanism remains unclear. Lower resistance to HSV-1 infection was shown in mice deficient in Ube2m or RNF11 (97).

Studies suggest that the acetylation of cGAS could have different outcomes, such as enhancement of its DNA-binding ability (98), suppression of cGAMP synthesis (99), hindrance of cGAS-dependent apoptosis (100) and amplification of inflammation in Systemic Lupus Erythematosus (101), while deacetylation of cGAS leads to its activation (100). Interestingly, the acetylation of cGAS in HFF cells was observed particularly in the nucleus and the perinuclear region, which increased upon *de novo* HSV-2 infection at early time point, thereafter reduced at later time points (**Figure 2B** and **Figures 5C, D**). Thus, further investigation is warranted to elucidate the exact nature, mechanism and effect of this dynamic PTM on cGAS activity and signaling during HSV-2 infection.

E3 Ub ligase Ring finger protein 185 (RNF185) specifically forms K27-linked polyubiquitin chain at K173 and K384 of cGAS, promoting its enzymatic activity (102). TRAF6 also polyubiquitinates cGAS upregulating type I IFN signaling (103). Contrastingly, TRIM56 – mediated monoubiquitination at K335 of cGAS C-terminal domain significantly increases cGAS dimerization, DNA-binding and cGAMP production, essential during HSV-1 infection in macrophages (104). TRIM41-mediated cGAS monoubiquitination also upregulates its activity and cGAMP synthesis (105). Additionally, cGAS K414 is constitutively K48-linked ubiquitinated, leading to degradation through p62-mediated autophagy-lysosomal pathway, rather than via the ubiquitin–proteasome system. Upon viral infection, TRIM14 promotes cGAS enzymatic activity, while recruiting the deubiquitinase USP14, which cleaves K48linked polyubiquitin chain of cGAS, disrupting the cGAS-p62 interaction and preventing autophagic degradation of cGAS (106). USP27x and USP29 enzymes similarly mediate deubiquitination of cGAS, promoting its stability (107, 108). Recently, K-63-linked polyubiquitylation of cGAS at K414 was reported to inhibit its DNA binding ability, the cGAMP production, and downstream immune response. This PTM was mediated by MARCH8 (also known as MARCHF8, c-MIR, and RNF178), which interacted with the enzymatically active core of cGAS through its conserved RING-CH domain. March8-deficient mice were shown to be less susceptible to HSV-1 infection (66). However, most reports on cGAS ubiquitination have focused on its cytoplasmic function, and whether nuclear cGAS undergoes specific ubiquitination modification, particularly during HSV-2 infection, has not been studied. Importantly, ours is the first report of K-63-linked polyubiquitination of cGAS in HFF cells, both in the nucleus as well as the cytoplasm, which was observed in uninfected condition, and showed dynamic pattern upon infection (**Figures 3A**, **5A, B**). The whole cell lysates showed that this PTM of cGAS reduced post infection (**Figure 2A**). Moreover, while K-63 ubiquitination of cGAS in the nucleus reduced from that in uninfected condition towards early and later time points post infection, it simultaneously increased in the cytoplasm through these time points (**Figures 3A**, **5A, B**). Deeper investigation is warranted to elucidate the mechanism, the host and viral factors involved in governing this dynamic pattern of K-63 linked polyubiquitination of cGAS in HFF cells during HSV-2 infection, as well as its implication on cGAS innate functions.

In conclusion, our study of the innate responses elicited during *de novo* HSV-2 infection in HFF cells revealed that the IFN-β response is biphasic. During the initial IFN-β response, cGAS associates with IFI16 in the nucleus, which is essential for the ability of cGAS to sense the nuclear input herpes viral DNA, whereas the late response is IFI16-independent, but cGAS-dependent, wherein cGAS senses the newly synthesized viral DNA in the cytoplasm. Thus, a complex cooperative role between IFI16 and cGAS during *de novo* HSV-2/1 infection was uncovered. Further, we also found that cGAMP is produced in the nucleus early during infection to elicit a rapid early response, thus extending the innate sensing function of cGAS and its enzymatic activity to the nucleus. Moreover, we also report the plausible role of cGAS in Caspase-1 cleavage during IFI16 inflammasome in the nucleus and NLRP3 inflammasome in the cytoplasm, at early and late times respectively, post HSV-2 infection in HFF cells. Interestingly, the IFI16 acetylation which is critical for inflammasome formation, is cGAS independent, suggesting that it is not sufficient for active Caspase-1 production, thus also suggesting a multifactorial regulation of Caspase-1 cleavage. The regulation of cGAS innate functions probably involves the dynamic K-63 linked polyubiquitination and acetylation. Further, it is possible that activated Caspase-1 mediates suppression of cGAS activity during infection. Furthermore, the interaction of STING with NLRP3, ASC and pro-Caspase-1 speculates STING-mediated NLRP3 inflammasome formation at early time point post infection. Moreover, the dynamic interaction of cGAS and Beclin-1 in the nucleus and in the cytoplasm, as well as the STING-LC3 interaction, suggests possible crosstalk between type I IFN and autophagy pathways during HSV-2 infection in HFF cells. Interestingly, the interaction of cGAS with γH2AX in the nucleus later during HSV-2 infection, suggests a possible role of cGAS in DDR. Importantly, the HSV-2 ICP27 interacts with STING early during infection, suggesting an immune evasion mechanism of the virus. Importantly, we observed dynamic cGAS acetylation and unique K-63 polyubiquitination of cGAS in HFF cells, the latter showing opposite patterns in the nucleus and the cytoplasm during HSV-2 infection. While deeper investigations into the cGAS-mediated inflammasome activation and the magnitude of its effect on type I IFN production were out of the scope of this study, it will be elucidated in our future studies. Similarly, the mechanism of STING-NLRP3 axis, specifically during HSV-2 infection needs to be described. Our further investigations will also reveal the structural basis, mechanism and role of the cGAS-STING interaction during HSV-2 infection. Further, deeper investigations are required to understand the exact mechanisms of cGAS and STING-mediated autophagy and the role of cGAS in DDR during HSV-2 infection, and the implications of these pathways on type I IFN and inflammasome responses. Our findings pave the way for further studies to define the cytoplasmic and nuclear discriminative sensing nature of the cGAS molecule. Deeper investigations are warranted to elucidate the role of various co-factors, PTMs and intricate crosstalk with various innate pathways in orchestrating the regulation of cGAS sensing, activity, sub-cellular localization, interactions and turnover, and how these mechanisms are manipulated by the virus for immune evasion. Further, single-cell omics studies could decipher as to what fraction of the total cGAS in the cell undergoes a particular regulatory event during the course of immune response. These dynamic spatial-temporal regulations also have important implications for other DNA sensors such as IFI16, which coordinate with cGAS. Our next study will undertake further investigations into other plausible functions of cGAS and IFI16, such as in the regulation of virus and host gene expression.

## Data Availability

The original contributions presented in the study are included in the article/supplementary material. Further inquiries can be directed to the corresponding author.
